# Modeling the relationship between neuronal activity and the BOLD signal: contributions from astrocyte calcium dynamics

**DOI:** 10.1038/s41598-023-32618-0

**Published:** 2023-04-20

**Authors:** Federico Tesler, Marja-Leena Linne, Alain Destexhe

**Affiliations:** 1 CNRS, Paris-Saclay Institute of Neuroscience (NeuroPSI), Paris-Saclay University, 91400 Saclay, France; 2 Faculty of Medicine and Health Technology, Tampere University, 33720 Tampere, Finland

**Keywords:** Computational neuroscience, Glial biology, Neural circuits, Neuro-vascular interactions

## Abstract

Functional magnetic resonance imaging relies on the coupling between neuronal and vascular activity, but the mechanisms behind this coupling are still under discussion. Recent experimental evidence suggests that calcium signaling may play a significant role in neurovascular coupling. However, it is still controversial where this calcium signal is located (in neurons or elsewhere), how it operates and how relevant is its role. In this paper we introduce a biologically plausible model of the neurovascular coupling and we show that calcium signaling in astrocytes can explain main aspects of the dynamics of the coupling. We find that calcium signaling can explain so-far unrelated features such as the linear and non-linear regimes, the negative vascular response (undershoot) and the emergence of a (calcium-driven) Hemodynamic Response Function. These features are reproduced here for the first time by a single model of the detailed neuronal-astrocyte-vascular pathway. Furthermore, we analyze how information is coded and transmitted from the neuronal to the vascular system and we predict that frequency modulation of astrocytic calcium dynamics plays a key role in this process. Finally, our work provides a framework to link neuronal activity to the BOLD signal, and vice-versa, where neuronal activity can be inferred from the BOLD signal. This opens new ways to link known alterations of astrocytic calcium signaling in neurodegenerative diseases (e.g. Alzheimer’s and Parkinson’s diseases) with detectable changes in the neurovascular coupling.

## Introduction

Functional magnetic resonance imaging (fMRI) has become one of the leading neuroimaging techniques in neuroscience. fMRI methods rely on the coupling between local neuronal activity and cerebral blood flow (CBF)^1,2^. In normal conditions an increase in neuronal activity is followed by an increase in CBF, which supplies the necessary oxygen and glucose to sustain the cerebral metabolism, a process known as *functional hyperemia*. In a healthy brain, the increase in oxygen supply overpasses the oxygen demand generating an increase in the local level of blood oxygenation, which is captured by the BOLD (Blood-Oxygen-Level-Dependent) signal^3^. Although the neurovascular coupling has been extensively demonstrated, the mechanisms behind it remain under discussion.

It is currently believed that glutamatergic synapses may play a central role in functional hyperemia^2,4,5^, inducing an increase of CBF via two main signaling pathways: a direct neuron-vascular pathway and a neuron-astrocyte-vascular pathway^6,7^. In both pathways, a glutamate-induced increase in intracellular calcium concentrations (in neurons or astrocytes) triggers the production and release of a variety of vasomodulators which generate a dilation of nearby arterioles and an increase in CBF. Among these vasomodulators we find potassium (K$^+$) and hydrogen (H$^+$) ions, prostaglandins (PGs), epoxyeicosatrienoic acid (EET) and nitric oxide (NO)^2,7,8^.

The relative relevance of these different signaling pathways is still under study. In particular, the role of astrocytes in the neurovascular coupling has been in the center of a controversy during the last decade. Some early studies suggested that astrocytic response was too slow to account for functional hyperemia^9,10^, which raised doubts about the participation of astrocytes in the coupling. However, due to experimental limitations, only somatic responses were measured in these early studies. It was later shown that a fast response can be observed in astrocytic processes and end-feet (from where vasodilators are thought to be released) and can be correlated with the initiation of the vascular response^11–13^. Thus, while a slow (or null) response may occur at the soma level, a fast response at astrocytic micro-domains is likely associated with the activation of the vascular response. Although further studies are necessary, the role of astrocytes in the neurovascular coupling is increasingly accepted^14^. The actual mechanism behind neurovascular coupling is likely a combination of direct neuron-vascular and neuron-astrocyte-vascular pathways, with the role and relevance of these pathways depending on the brain region, cell-type and even brain-state^2,6,7^.

Despite the different sources and effects on the vasculature, one feature that the different coupling mechanisms seem to share is that they are usually associated with some type of fast intracellular calcium (Ca$^{2+}$) activity. The relation between calcium signaling in the brain and the vascular response has started to be further explored in experiments during the last few years. Calcium activity in neuronal-dendrites, neuronal-body and astrocytes has been analyzed in relation with the vascular response^6,11,15–17^. Thus, the experimental evidence seems to suggest that calcium signaling (in neurons or astrocytes) may play a central role in the information processing and transmission between the neuronal and vascular systems. There is however little understanding on how the processing of information is performed by the calcium signal and how it operates on the formation of the BOLD signal.

The goal of this work is to study the function of astrocytic calcium activity in functional hyperemia. To perform our study we introduce and analyze a biologically plausible model of the neurovascular coupling which incorporates recent experimental findings and modelling tools at the three levels of the system (neuronal, astrocytic, vascular). Following experimental evidence we will consider the (calcium-mediated) release of the prostaglandin PGE2 by astrocytes as the principal vasomodulator acting in the coupling^7,8^. Neuronal (and synaptic) activity will be modeled via a recently developed mean-field description of a network of Adaptive Exponential(AdEx) integrate-and-fire neurons^18^, which allows us to explicitly integrate neuronal adaptation. The vascular system will be described by a novel formulation that relates arteriole volume to cyclic-AMP concentration, together with the classical Balloon model for the BOLD signal^19,20^.

Starting from a relatively detailed description we will focus on fundamental aspects of the fMRI phenomenology (such as main experimental protocols, the Hemodynamic Response Function, the linearity of the coupling, the post-stimulus undershoot and the effects of neuronal adaptation) and we will analyze the role of the calcium signal in these processes. Previous studies with models of the neuron-astrocyte-vascular interaction exists^21–24^, but to the best of our knowledge, this is the first work that accounts for the above experimental observations of fMRI.

We show that information transmission by the calcium signal operates mainly via frequency coding with a small contribution of amplitude modulation. We also show that in our model calcium signaling is responsible for both linear and non-linear aspects of the neurovascular coupling. In Section “Linear coupling and the hemodynamic response function” we show that a calcium-driven Hemodynamic Response Function (HRF) can be generated and that it is equivalent to the one observed experimentally. We corroborate this by comparing our results with the canonical HRF and we reproduce our simulations using the HRF formalism. We also show that the calcium activity is a better predictor of the BOLD response within this context. In Section “BOLD undershoot and calcium dynamics” we study situations where the BOLD signals can become negative (post-stimulus undershoot), a well-known feature observed in fMRI experiments. We show that an undershoot in the calcium activity can cause an undershoot in the BOLD signal via a decrease in CBF. Finally, in Section Adaptation effects we study the effects of neuronal adaptation on the calcium and BOLD signals. We show that the calcium signal can capture the effects of neuronal adaptation via calcium-spike frequency adaptation and transmit it to the BOLD signal. In particular we show that adaptation may play a major role in the post-stimulus undershoot, which further stresses the usefulness of adopting a mean-field formalism where adaptation is present. We end by showing how information about the incoming and recurrent neuronal activity can be extracted from the BOLD signal in our modeling framework.

The current work is focused on calcium signaling in astrocytes, however the results of our study can be extended to other calcium-driven neurovascular pathways as we will discuss along the paper.

## The model

**Figure 1:**
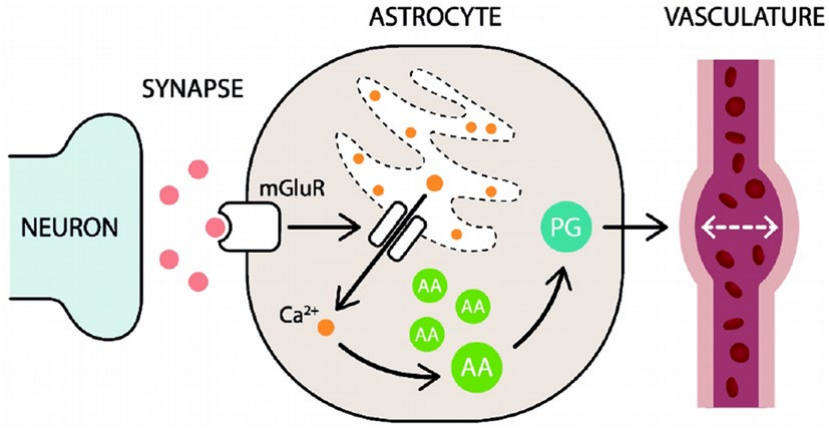
Diagram of the model of neurovascular coupling. We consider a feedforward system that starts at the neuronal level. Glutamate spilled from the synaptic clefts toward the perisynaptic space activates, through metabotropic glutamate receptors (mGluRs), the calcium (Ca$^{2+}$) signaling cascade in astrocytes. The increase in cytosolic Ca$^{2+}$ levels in astrocytes induces the production and release of vasomodulators (prostaglandin, PG) derived from free arachidonic acid, leading to the variations of the arteriole volume.

The model of the neurovascular coupling that we propose consists in a feedforward system comprising the neuronal activity, the calcium dynamics in astrocytes and the vascular response. A diagram of the model is shown in Fig.  1. Neuronal activity will be described via a mean-field model of a network of AdEx neurons. Calcium dynamics in astrocytes will be described via the traditional Li-Rinzel model with recent improvements by De Pittà et al. (2009) to incorporate IP3 dynamics by glutamate activation^25,26^. The Li-Rinzel model describes the flux of $Ca^{2+}$ between the cytosolic and the endoplasmic reticulum (ER) of the cell, which is known to be the main source of calcium activity in astrocytes^7,8^. To describe the communication between astrocytes and the vascular system we will model the production of the prostaglandin PGE2 (via the arachidonic acid (AA) cascade) and the action of this vasomodulator on arteriolar smooth-cells which induces the changes in cerebral blood flow. Finally, the BOLD signal generation is modeled via the Balloon model^19^. The details of the model are introduced in the following.

### Mean-field neuronal model and synaptic release

To model the neuronal activity we will consider a network of adaptive exponential-integrate-and-fire neurons (AdEx)^27^. We will consider a directed network made by 10000 AdEx neurons, with 80$\%$ of excitatory and 20$\%$ of inhibitory neurons. Neurons in the network are randomly connected with probability $p=5\%$ and interact via conductance based synapses (see Supp. Inf.Materials and Methods for details). We will focus on the mean activity of the population. The mean-field equations for the AdEx network are given to a first-order by^18^:

1 $$\begin{aligned} T\frac{d\nu _{e,i}}{dt}= & {} F_{e,i}(W,\bar{\nu }_e,\nu _i)-\nu _{e,i} \end{aligned}$$

2 $$\begin{aligned} \frac{dW}{dt}= & {} -\frac{W}{\tau _w} + b\nu _e + a(\mu _V(\bar{\nu }_e,\nu _i,W)-E_L) \end{aligned}$$

where $\nu _{e,i}$ is the mean neuronal firing rate of the excitatory and inhibitory population respectively, *W* is the mean value of the adaptation variable, *F* is the neuron transfer function (i.e. output firing rate of a neuron when receiving excitatory and inhibitory inputs with mean rates $\nu _e$ and $\nu _i$ and with a level of adaptation W), *a* and *b* are the sub-threshold and spiking adaptation constants, $t_w$ is the characteristic time of the adaptation variable and *T* is a characteristic time for neuronal response (we adopt $t_w=1$ s and $T=5$ ms ). Values for all the parameters are given in Supp. Inf.Table 1. The stimulation of the network is simulated by an (excitatory) external drive $\nu _{ext}$, which represents the input from a nearby neuronal population and so $\bar{\nu }_e=\nu _e+\nu _{ext}$. The network exhibits no spontaneous activity for the parameters used. Derivation of the mean field equations can be found in Ref.^18^.

Synaptic glutamate release can be straightforwardly estimated from the mean-field model. Glutamate concentration ([Glu]) is proportional to the mean conductance of excitatory synapses (*s*) obtained from the (conductance-based) mean-field model^18^, $s=\nu _e pN_eQ_e\tau _e$, where $N_e$ is the number of excitatory neurons, $Q_e$ is the excitatory quantal conductance (conductance change induced by a single spike) and $\tau _e$ is the conductance decay time (see Supp. Inf. for details and Supp. Inf. Table 1 for the corresponding parameter values). Thus, [Glu]=*gs*, where *g* is a proportionality constant. For a better analysis, we will separate the contribution of the recurrent excitatory synaptic activity from the one of the external excitatory input. Thus, the mean glutamate concentration in the synaptic cleft is given by:

3 $$\begin{aligned}{}[Glu]= (g_r \nu _e + g_{ext} \nu _{ext})pN_eQ_e\tau _e \end{aligned}$$

where $g_{r/ext}$ is the proportionality constant accounting for the contribution to glutamate release of the recurrent and external excitatory synaptic activity respectively.

We notice that the activity of inhibitory neurons may also be involved in the neurovascular coupling, for example via release of nitric oxide which acts as a vasodilator. Nevertheless, it is currently believed that excitatory synaptic activity dominates the overall ATP cost of neural signaling for which it is expected excitatory activity to be a strong driver of vascular response^28,29^. In addition, our study is focused on the neuronal-astrocyte interaction which is mainly driven by synaptic glutamatergic release. For these reasons we will focus on the role of synaptic release of excitatory neurons.

### Astrocyte calcium dynamics and arachidonic acid cascade

In this section we present the model used to describe the intracellular calcium dynamics and vasomodulator production by astrocytes. In our model we assume that part of the neuronal glutamate released to the synaptic cleft spills toward the perisynaptic space and activates mGluRs in nearby astrocytes, which triggers the astrocytic calcium signaling. We notice at this point that the exact location and path of the calcium signal within the astrocyte in response to neuronal activity is still under discussion, being the signal likely initiated in astrocytic processes and propagates towards the end-feet where vasodilators are released^11,30^ (although, direct initiation at the end-feet could also be possible^13^). In any case, the propagation and/or initiation of the calcium signal within the astrocyte occurs in times much shorter than the initiation of the vascular response, the former being in the order of tens of milliseconds^11,13^ and the latter in the order of seconds. In our model we will assume that the calcium signal starts at the process-level and propagates to the end-feet. As the time of propagation and/or initiation of the calcium signal is negligible compared to the time of initiation of the vascular response we will neglect any delay due to the signal propagation and assume that calcium transients occur simultaneously in the process and in the end-feet. Thus, we will assume that calcium concentration in both compartments can be described by a single variable $Ca^{2+}$, where we assume that the signal is preserved during the propagation.

To model the glutamate-induced calcium dynamics in the astrocyte we adopt a recent version of the Li-Rinzel model^25^ which incorporates a detailed dynamics of $Ca^{2+}$-dependent production and degradation of cytosolic inositol trisphosphate (IP3)^26,31^. In this model, the activation of mGluRs induces an increase of intracellular cytosolic IP3 that triggers the release of calcium from the endoplasmic reticulum (ER) towards the cytosol. This generates a transient increase in cytosolic Ca$^{2+}$ which is later reabsorbed by the ER. For details on the model see Refs.^25,26,31^ and Supp. Inf. Materials and Methods (Fig. S1–S2). The Li-Rinzel model equations for the cytosolic calcium concentration and IP3 gating (*h*) are given by^25^:

4 $$\begin{aligned} \begin{aligned} \partial _t C^{2+}&=J_C(Ca^{2+},h,IP3)+J_L(Ca^{2+})-J_P(Ca^{2+}) \\ \partial _t h&=\frac{h_\infty (Ca^{2+},IP3)-h}{\tau _h(Ca^{2+},IP3))} \\ \end{aligned} \end{aligned}$$

where $J_C$ , $J_L$ , and $J_P$ denote the IP3-mediated $Ca^{2+}$ -induced $Ca^{2+}$ -release from the ER ($J_C$ ), the $Ca^{2+}$ leak from the ER ($J_L$ ), and the $Ca^{2+}$ uptake from the cytosol back to the ER by serca-ER $Ca^{2+}$ /ATPase pumps ($J_P$ ) [141]. These terms are given by

5 $$\begin{aligned} \begin{aligned} J_C&=\Gamma _Cm_\infty ^3h^3(C_T-(1+\rho _A)Ca^{2+}) \\ m_\infty&=H(IP3,d_1)H(Ca^{2+},d_5)\\ J_L&=\Gamma _L(C_T-(1-\rho _A)C)\\ J_P&=O_PH(Ca^{2+},K_P)\\ h_\infty&=d_2\frac{IP3+d_1}{d_2(IP3+d1)+(IP3+d_3)Ca^{2+}}\\ \tau _h&=\frac{1}{O_2}\frac{IP3+d_3}{d_2(IP3+d1)+(IP3+d_3)Ca^{2+}}\\ \end{aligned} \end{aligned}$$:

where $\tau _h$ is the IP3 receptor (IP3R) deinactivation time constant and $h_\infty$ is the steady-state probability. Here $C_T$ is the total calcium concentration at the ER, $\rho _A$ is the ER-to-cytoplasm volume ratio and $\Gamma 's$, $d's$ and $O_2$ are constants. For a detailed explanation of the parameters see Supp. Inf. and ref.^31^. In future sections we will explore in particular the effect of the deinactivation time constant ($\tau _h$) in the calcium response relevant for the neurovascular coupling.

The increase of cytosolic Ca$^{2+}$ activates phospholipase A2, which induces the production of arachidonic acid (AA) from membrane phospholipids. This in turn leads to the production and release of vasomodulators derived from AA, in particular the vasodilator PGE2^2,7,8^. The production of AA and PGE2 will be modeled via Michaelis-Menten type of equations^21^:

6 $$\begin{aligned} \frac{dAA}{dt}= & {} -\frac{AA}{\tau _{AA}} + \frac{O_{AA}Ca^{2+}}{K_{AA}+Ca^{2+}} \end{aligned}$$

7 $$\begin{aligned} \frac{dPG}{dt}= & {} -\frac{PG}{\tau _{PG}} + \frac{O_{PG}AA}{K_{PG}+AA } \end{aligned}$$

where $\tau _{AA}$ and $\tau _{PG}$ are the time constants for AA and PGE2 respectively, $K_{PG}$ and $K_{AA}$ are the Michaelis constants and $O_{PG}$, $O_{cAMP}$ are the maximum rate of production of AA and PGE2 respectively. We note that this model describes a single cell activity. Vascular activation is most likely not carried out by a single astrocyte (or single astrocyte process) but by the many astrocytes linked with the local vasculature and neuronal population. Nevertheless, it has been seen that *in vivo* the activity of astrocytes tend to be restricted to independent single-cell activity^32–35^ (rather than intercellular calcium waves as seen in *in vitro* experiments^36^). Thus, we will adopt this single-cell model as a representative unit of the astrocytes associated with the same neuronal population.

Regarding the adoption of PGE2 as the main vasodilator in the coupling, we follow recent studies that suggest that the main part of the vascular dilation generated by astrocytes ($\sim$70$\%$) is driven by prostaglandins^7,37^. The rest of the dilation may be generated by other vasodilators such as EET, and both can be acting together in the coupling^8^. For simplicity in this study we have only considered PGE2, but the incorporation of vasodilators such as EETs can be envisioned for future extensions of our model.

Regarding the IP3-related calcium response, we notice that a variety of IP3R calcium channel subtypes have been detected in astrocyte fine processes^38^, although the specific subtypes of IP3 receptors potentially involved during fast calcium responses is still under discussion. In this work we assume a generic IP3R involved in the IP3-induced calcium dynamics. A detailed analysis of potential IP3R subtypes is out of the scope of this paper.

We point out that levels of calcium concentration in our model are intended to be in agreement with typical values observed in experiments, but these concentration can be adjusted without significant change in the results presented in the paper.

Finally, regarding the type of glutamate receptors acting in the calcium transient activation, one possible candidate is the mGluR5, although it has been observed that the expression of this mGluR subtype tends to reduce in adult animals^39,40^. More recently it has been also proposed that the mGluR3 subtype in combination with other Gq-coupled receptors can be acting behind the activity of calcium transients in astrocytes^41^. As we did for the IP3R, in this paper we assume a generic mGluR involved in the calcium signal activation, and a detailed analysis of mGluR subtypes is out of the scope of this paper.

### Vascular response and BOLD signal

We will assume that the vascular response starts at the arteriole level. We assume that PGE2 released from astrocytes binds to EP4 prostaglandin receptors on smooth cells of arterioles inducing an increase of cyclic adenosine monophosphate (cAMP) concentration in the smooth cells. This generates the activation of protein kinase A and a decrease in the phosphorylation of the myosin light chain. Contraction of arterioles has been proposed to be proportional to the concentration of phosphorylated myosin^22^. Following this line, we will assume that arteriole dilation is proportional to the concentration of cAMP. The equations describing the fraction of activated PGE2 receptors and the cAMP production in arterioles are:

8 $$\begin{aligned} \frac{dR_{PG}}{dt}= & {} -\frac{R_{PG}}{\tau _R}+O_R PG(1-R_{PG}) \end{aligned}$$

9 $$\begin{aligned} \frac{dcAMP}{dt}= & {} -\frac{cAMP}{\tau _{cAMP}} + O_{cAMP}R_{PG} \end{aligned}$$

where $R_{PG}$ is the portion of activated PGE2 receptors, *PG* is the concentration of PGE2, $\tau _{cAMP}$ is the relaxation time for cAMP, and $O_R$, $O_{cAMP}$ are constants. Normalized relative arteriole volume is given by:

10 $$\begin{aligned} CBV_{A}=1 + D_A\frac{(cAMP-cAMP_o)}{K_{VA}^2+(cAMP-cAMP_o)^2} \end{aligned}$$

where $CBV_A$ is the normalized change of arteriolar cerebral blood volume relative to its basal value, $cAMP_o$ is the basal concentration of cAMP and $D_A$ ,$K_{VA}$ are constants.

The relation between CBV and CBF is usually assumed to follow a power law. The value of the exponent has been observed to depend on the specific brain region and vasculature level^42,43^. In our model the relative change in cerebral blood flow evoked by changes in $CBV_A$ is given by:

11 $$\begin{aligned} CBF_{IN}=CBV_A^{\frac{1}{\alpha _A}} \end{aligned}$$

where $\alpha _A$ is a constant (a different power $\alpha _V$ is used for the venule blood volume $CBV_V$, see Supp. Inf. Materials and Methods).

Finally, to generate the BOLD signal evoked by the changes of CBF we will adopt the well-known Balloon model^19^. This model describes the change in dexohemoglobin concentration (which originates the BOLD signal) generated by an increase in CBF. A rough approximation of the BOLD signal obtained from the Balloon model in terms of CBF is given by^44^:

12 $$\begin{aligned} BOLD\approx k_1(CBF_{IN}-1) \end{aligned}$$

where $k_1$ is a constant. This approximation provides a good intuition of the expected BOLD response and is given here for reference, however it fails to reproduce some key features such as the post-stimulus undershoot related with slow venule volume relaxation (analyzed later in the paper). For all the simulations presented in this paper we use the full Balloon model as presented in the Supp. Inf. (Materials and Methods). For details on the Balloon model see Refs.^19,20^ and Supp. Inf. Materials and Methods.

## Results

In the following we present the results obtained from our model. First we analyze the time courses of the main variables in the vascular pathway and we reproduce some key experimental results from fMRI measurements. In particular, we show that our simulations can correctly reproduce the results of the two main experimental protocols used in BOLD imaging, namely i) event-related protocols and ii) block protocols. Then we proceed with the study of information coding and transmission in our system (Calcium coding and BOLD response); the analysis of the linear coupling and the Hemodynamic Response Function formalism (Linear coupling and the hemodynamic response function); the analysis of the undershoot of the BOLD signal in the context of the calcium pathway (BOLD undershoot and calcium dynamics); and the effects of neuronal adaptation on the calcium signal and the BOLD response (Adaptation effects).

### Event-related and block protocols

In this section we show how the calcium pathway for neuro-vascular coupling operates and we show that we can correctly reproduce the results from the main experimental protocols used in fMRI, which provides a first validation for our model.

Experimental protocols used for BOLD imaging are usually divided in two groups^1,45,46^: i) event-related protocols, where short single stimulus are presented with long inter-stimulus intervals (tens of seconds, equal or larger than typical BOLD response time-course); and ii) block protocols, where multiple stimuli are presented with very short inter-stimulus intervals, followed by a longer ’inter-block’ interval after which a new set (’block’) of stimuli is presented. In both protocols stimulus duration can be in the order of 4s or less. Variations and mixed-type protocols are also used. For a detailed analysis of the protocols and their advantages see Refs.^1,45–48^. We notice that these two protocols are related to fundamental features of the neurovascular coupling, which are the vascular response to an impulse stimulus and the response to a superposition of stimulus. The analysis of of these two features constitutes an important part of this work.

**Figure 2:**
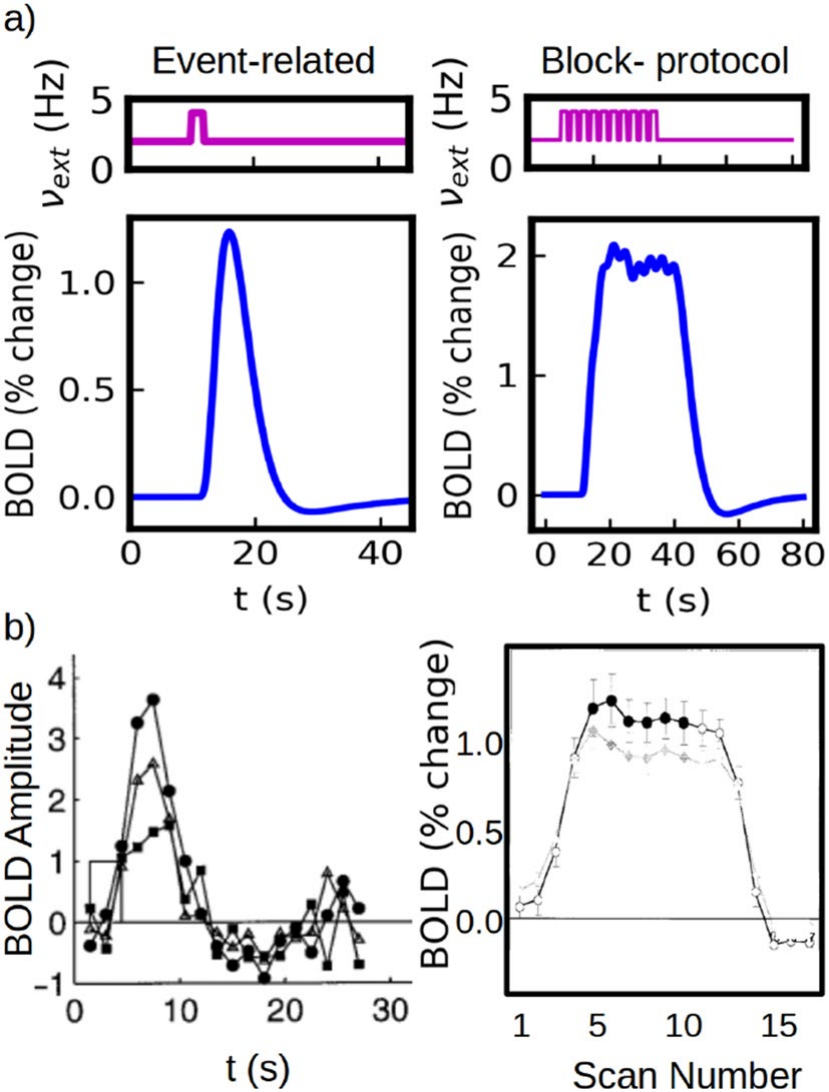
Standard experimental protocols for fMRI measurements. (**a**) Simulations of the event-related and block protocols. The event-related case consists in the application of a single stimulus of 2s. The block-protocol consists in the application of 10 stimuli of 2 seconds each, separated by an inter-stimulus interval of 1 second. (**b**) Experimental results for the two standard protocols. Left: results from an event-related protocol for three different stimulus strength (stimulus duration of 3 seconds), adapted from Ref.^49^. Right: results from a block protocol with 2.5 seconds stimulus and an inter-stimulus interval of 0.5 seconds, adapted from Ref.^48^.

The results of our simulations for the two protocols are shown in Fig. 2. Comparison with experimental results are shown in panel (b). Except otherwise indicated, the stimulus used in the paper will consist in a pulse of the external input $\nu _{ext}$ as shown in the top panel of Fig. 2a (the width of the pulse corresponds to the duration of the stimulus).

The response obtained for a single (short) event is usually known as the Hemodynamic Response Function (HRF) and corresponds to the minimum BOLD response in functional hyperemia. The same response is obtained for any stimulus of shorter duration surpassing a certain threshold in time and intensity. In our model, this minimum response corresponds to the activation of a calcium-spike as we will show later. As we see from the figure, our simulations can capture main features of the HRF: i) a lag of $t_{ON}\approx$ 2 seconds between the application of the stimulus and the activation of the response; a lag of $t_{peak}\approx$ 5 seconds between the application of the stimulus and the peak of the response; a maximum amplitude that range between 1$\%$ and 2$\%$ (measured as percentage change from the basal level); a marked post-stimulus undershoot; and a total duration in the order of few tens of seconds. The HRF is analyzed in detail in Section “Linear coupling and the hemodynamic response function”. From the simulation of the block protocol we see that we can correctly capture the response to the superposition of events (further analysis on this is developed in Section “Linear coupling and the hemodynamic response function”).

**Figure 3:**
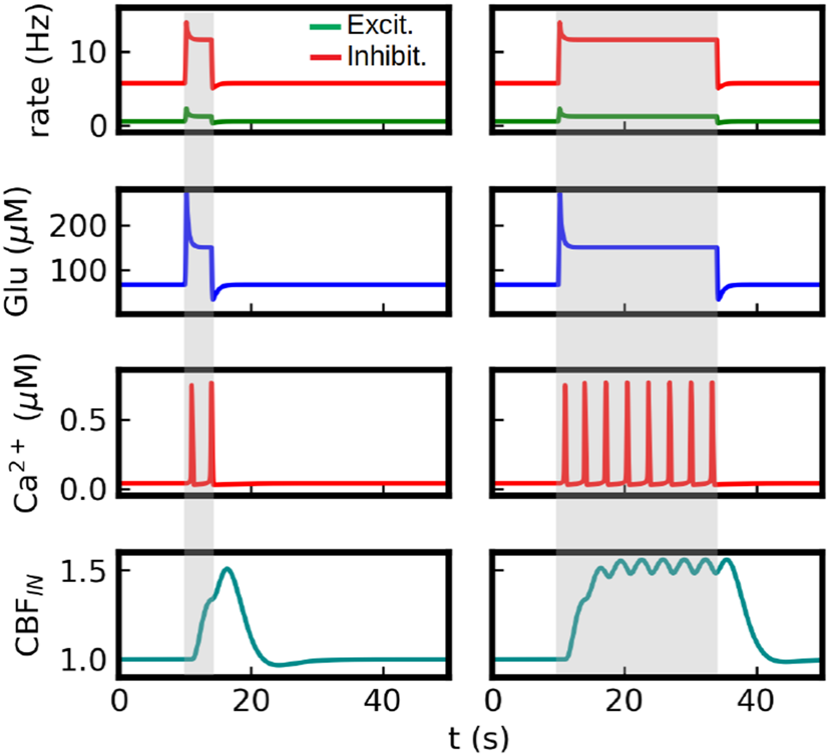
Time course of main model variables for a stimulus of 4 seconds (left) and 24 seconds (right). The interval of stimulus application is indicated by the grey area. The figure illustrates the calcium pathway of neurovascular coupling in the model. The stimulus (represented as an increase in the external input $\nu _{ext}$, not shown) generates an increase in neuronal activity (top panel) which induces a rise in the level of perisynaptic glutamate concentration (second panel). Glutamate triggers the activation of calcium dynamics in astrocytes which is characterized by brief calcium ’spikes’ as seen in the figure (third panels from the top). This leads to the production and release of the vasomodulator PGE2 (not shown, see Supp. Inf. Materials and Methods) which dilates the nearby arteriole and increases the flow of blood into the tissue (bottom panel). The simulations correspond to $\nu _{ext}$=4 Hz.

To provide a better understanding of the mechanism of the BOLD generation in our system we show in Fig. 3 the time course of the main variables of the model for stimuli of two different duration (4s and 24s). In Fig. 3 (top panels) we see that a fast neuronal response is generated after the application of the stimulus, which exhibits a marked overshoot given by the strong adaptation effect. This neuronal response generates an increase in the perisynaptic glutamate concentration (second panels from top) that triggers the activation of the calcium dynamics in the astrocyte (characterized by calcium-spikes as seen in Fig. 3 third panels from top). The Ca$^{2+}$ activity leads to the increase in cerebral blood flow (CBF$_{IN}$, bottom panels) driven by the release of the vasomodulator PGE2 and the increase in arteriole volume (not shown, see Supp. Inf. Materials and Methods for the time course of the remaining variables). We notice that CBF$_{IN}$ is linked to the arteriole volume CBV$_{A}$. Timing and size differences between arteriole and venule volume changes are also correctly captured by our simulations and are shown in the Supp. Inf. Materials and Methods. As we will see in the next section, the information about the external stimulus is coded in terms of the frequency and amplitude of calcium spikes.

In the simulations presented in this section we set $g_{ext}=0$ in Eq. (3), meaning that the glutamate release is proportional to the local excitatory activity $\nu _e$. Nevertheless, we notice that, for the level of neuronal adaptation that we use, the asymptotic activity of the population is mainly driven by the external input with a small contribution of the recurrent activity (and $\nu _e$ is nearly proportional to $\nu _{ext}$, as seen in the next section). The recurrent activity is only relevant during a short period after the stimulus onset (neuronal overshoot), defined by the characteristic time of the adaptation variable ($t_w$). As this characteristic time is short compared to the calcium response, then the BOLD response in these simulations is mainly driven by the asymptotic neuronal activity (and thus by the external input). Thus, the results presented here for the BOLD signal are nearly independent of the $g_{r/ext}$ values we use. We will use $g_{ext}=0$ through the paper, except otherwise indicated. As we will see in Section “Adaptation effects”, when $t_w$ is comparable to the time of the calcium response, then the adaptation effects have a relevant impact in the BOLD response and, in particular, we will see that information of the recurrent neuronal activity can be extracted from the post-stimulus undershoot of the BOLD signal.

### Calcium coding and BOLD response

**Figure 4:**
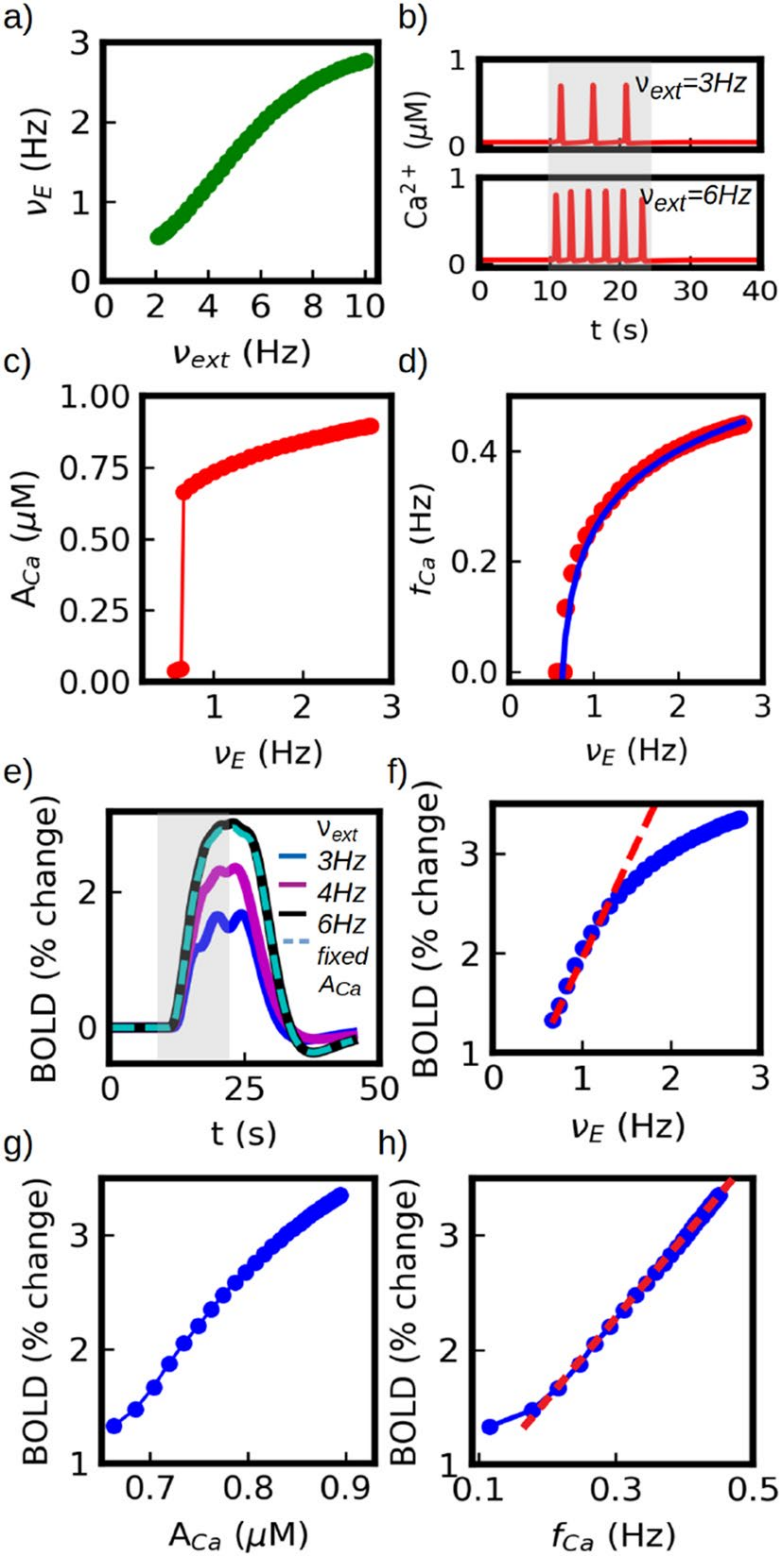
Neuronal and calcium coding of an external stimulus. (**a**) Average firing rate of the excitatory neuronal population as a function of the external stimulus intensity (in terms of the external excitatory rate $\nu _{ext}$). (**b**) Astrocytic calcium activity for two values of $\nu _{ext}$ . (**c**, **d**) Amplitude and frequency of calcium-spikes as a function of excitatory firing rate. External stimuli are first codified by neuronal activity which modulates the amplitude and frequency of the calcium response. In panel (**d**) we show a fit with a logarithmic function (solid blue line, see main-text). (**e**) BOLD response for different stimulus intensity (for a comparison with experimental results see Fig. 2. (**b**) In dashed line we show the results for $\nu _{ext}$=6 Hz with calcium-spike amplitude limited to A$_{Ca}$=0.65$\mu$M for arachidonic acid production, indicating that the main increase in BOLD response is not driven by the spike-amplitude. Frequency-coding accounts for about 95$\%$ of the variation. (**f**) BOLD response as a function of excitatory activity. A linear fit is shown in red dashed line, which describes $\sim$75$\%$ of the total BOLD variation. (**g**, **h**) BOLD response to stimuli intensity parameterized in terms of calcium-spike amplitude and frequency. A linear fit is provided in panel (**h**) (dashed red line). For a comparison of panel (**g**) with experimental results see Ref.^17^.

In this section we analyze how the information about the input stimulus and the neuronal activity is codified and transmitted by the calcium signal to the vascular system. In particular, we focus on how the intensity of the stimulus (represented by the external input $\nu _{ext}$) is codified. We separate the coding of the input stimulus into two parts: the first coding performed by the neuronal activity and the subsequent coding of the neuronal activity performed by the calcium signal. In Fig. 4a we show the response of the excitatory neuronal population as a function of the external input $\nu _{ext}$ , obtained from the mean field model (Eqs. 1 and 2). The curve corresponds to the steady value of the neuronal activity, after $\nu _{E}$ and *W* have reached an equilibrium. The response exhibits a non-linear behaviour with signs of saturation for the highest inputs. This neuronal activity is then sensed by the astrocyte and codified by means of the intracellular calcium signal. We notice that the neuronal-astrocyte communication is made through glutamate release, which in our model operates linearly on the curve of Fig. 4a. In Fig. 4b we show the time course of the calcium signal for two different inputs ($\nu _{ext}$=3 and 6 Hz) and in panels (c),(d) we show the evolution of the frequency ($f_{Ca}$) and amplitude ($A_{Ca}$) of the calcium signal as a function of the neuronal (excitatory) firing rate ($\nu _E$). It can be seen that the neuronal activity is codified by the calcium signal in terms of both its frequency and amplitude. We observe that the signal exhibits a threshold for calcium-spike generation, under which no spike is observed. Over the threshold, both amplitude and frequency are monotonic increasing functions of the firing rate (for a detailed analysis of the calcium dynamics and the coding modes see Supp. Inf. and Ref.^26^). The modulation of the frequency and amplitude by the neuronal activity is made via the control of the IP3 production rate by glutamate concentration as given in the modified Li-Rinzel model (see Ref.^31^ and Supp. Inf.).

The frequency-input relation of the calcium response can be correctly described by a logarithmic function. A similar functional description has been proposed before for analogous gating dynamics^50,51^. We show in Fig. 4d the results of a fit over the $f_{Ca}-\nu _E$ curve with the function $f(x)=Aln(\frac{x-x_o}{B})$, where $A=0.12$, $x_o=0.58$ and $B=0.05$. We notice that the expression used for the fit is purely phenomenological. Nevertheless it provides a quantitative description of the calcium frequency response that will be of use for later analysis in the paper. We notice in addition that the maximum value of $f_{Ca}$ is mainly defined by the deinactivation time of the IP3 gated channels that establishes a refractory time for calcium-spike generation. An analysis of the frequency-range is given in Section “Model robustness and further comparison with experimental results”.

Finally, the calcium signal is transmitted to and decodified by the vascular system through the arachidonic acid avalanche and cAMP pathway as explained in the previous sections. In Fig. 4e. we show the BOLD response for three different inputs ($\nu _{ext}$=3, 4 and 6 Hz). We see that the variation in the input performs basically a re-scale in the amplitude of the BOLD signal.

In Fig. 4f we show the amplitude of the BOLD response as a function of the neuronal activity $\nu _{E}$. A linear fit is shown in red dashed line, which describes nearly 75$\%$ of the total BOLD variation in our simulations. After this linear regime, a saturation in the response is observed. In Fig. 4g,h we show the BOLD response to input intensity parameterized in terms of calcium-spike amplitude and frequency. A similar relation between BOLD and the amplitude of astrocytic $Ca^{2+}$ signal has been seen in experiments^17^. Nevertheless, we notice that the change in BOLD response in our simulations is mainly driven by the frequency of the calcium signal and not by its amplitude. We show this in Fig. 4e (dashed line) by imposing a fixed $A_{Ca}$ for arachidonic acid production. The change in $f_{Ca}$ accounts for more than $95 \%$ of the BOLD amplitude variation, while the other less than 5$\%$ is given by the change in $A_{Ca}$. In addition, we see that the BOLD-$f_{Ca}$ relation is approximately linear, except near the threshold where the linearity is lost.

The threshold for calcium-spike generation observed in the model is related with the balance between $Ca^{2+}$ release and re-uptake from the ER of the cell, together with the IP3 activation of calcium channels (see Eq. 4). Given the non-linearity of the system, this threshold has a strong dependency on the maximum rates of glutamate-dependent IP3 production and on the rate of IP3-induced calcium realease from endoplasmic reticulum. Dysfunctions in the rate of calcium release (for example, by variation in the density of IP3 receptors in the membrane of the endoplasmic reticulum) may lead to an increase in the threshold level of glutamate concentration for calcium-spike activation, meaning that a higher synaptic activity would be necessary to evoke a significant hemodynamic response. Further increase in the threshold level would lead to a decoupling of the neurovascular response (an example of such a dysfunction is presented in the Supp. Inf.).

### Linear coupling and the hemodynamic response function

**Figure 5:**
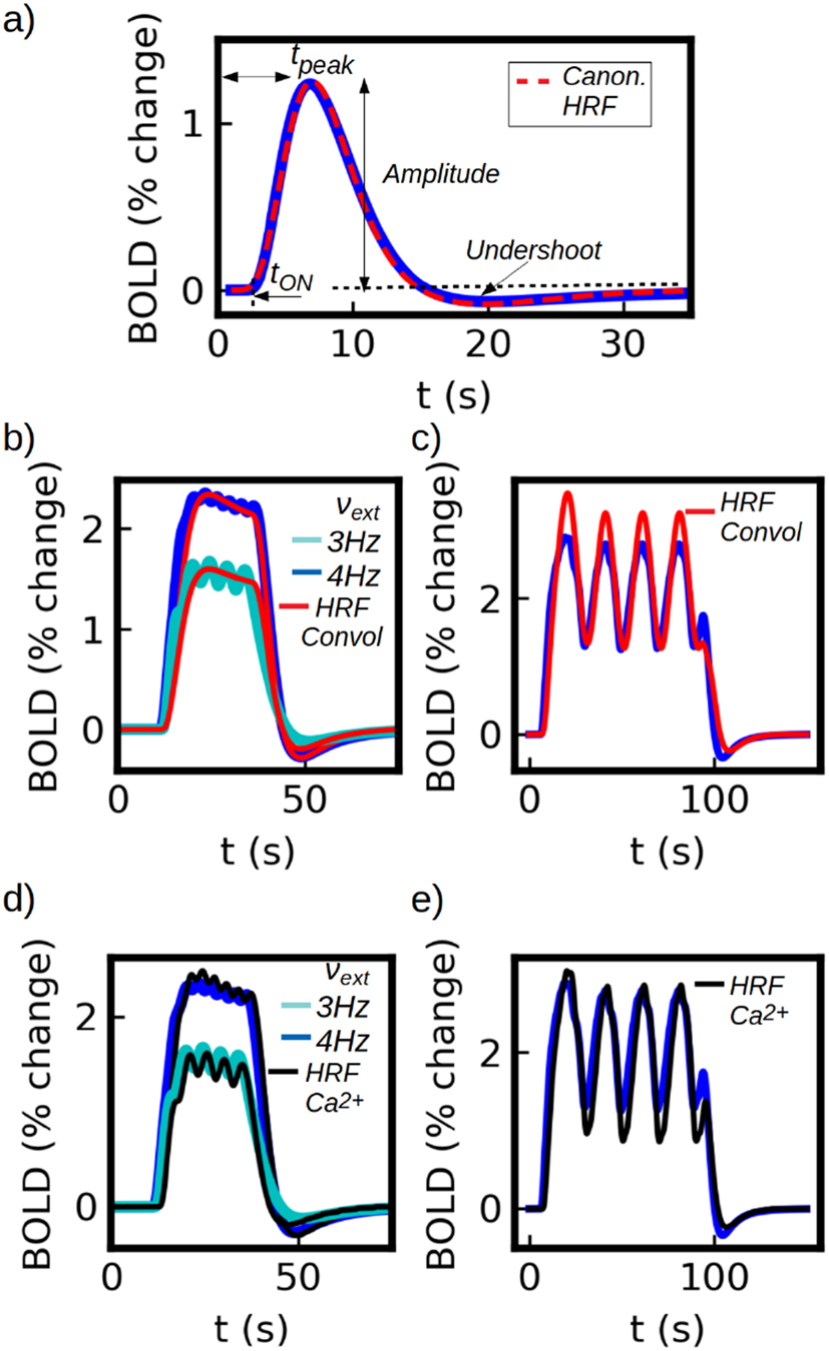
(**a**) Hemodynamic Response Function (HRF) obtained from our model, corresponding to a stimulus of 2 seconds and $\nu _{ext}$=4 Hz (blue solid line) . A fit with a double-gamma canonical HRF is shown (red dashed line), HRF$_C=(\frac{t}{d_1})^{a_1}e^{-(t-d_1)/b_1}-c(\frac{t}{d_2})^{a_2}e^{-(t-d_2)/b_2}$. The coefficients from the fit are $d_1=6$s, $a_1=6$,$b_1=1s$,$c=0.07$, $d_2=18$s, $a_2=12$,$b_2=1.5$s, in good agreement with experimental values^52,53^. In panel (**b** and **c**) we show the BOLD response together with the result from the convolution of Eq. (13). In panel (**b**) we show the results for a 24 seconds stimulus with $\nu _{ext}$=3 and 4 Hz. In panel (**c**) we show the results for a sinusoidal stimulus centered at $\nu _{ext}$=4.2 Hz and amplitude of 1.6 Hz. We see that the results from the convolution fails to reproduce the simulations for the higher values of the input as the system moves away from the linear regime. In panel (**d** and **e**) we show the BOLD response estimated via the convolution of the HRF with the calcium activity (for the same stimuli as panels (**b**, **c**) respectively). From panel (**e**) we see that the results from the convolution can reproduce the simulations except for the lower values of the input (close the threshold for calcium activation) as the system moves away from the linear regime.

The BOLD response shown in Fig. 2a for a single short (<4s) stimulus is known as the Hemodynamic Response Function (HRF). In fMRI experiments it is usually assumed that the neuronal and vascular activity are linearly coupled, which has shown to be valid in a certain parameter range^6,49^. Assuming that the coupling is linear and time invariant, it is possible to write the vascular (BOLD) response as^49^:

13 $$\begin{aligned} BOLD=\int HRF(t-\tau )r(s,t) \end{aligned}$$

where *r*(*s*, *t*) is the time course of neuronal activity of the local population, being *s* the intensity of the stimulus or other stimulus parameter under study. In this section we study the HRF obtained from our model and the validity of the linear analysis. The HRF obtained for a stimulus of 2 seconds and $\nu _{ext}$=4 Hz is shown in detail in Fig. 5a. Main features of the HRF (indicated in the figure) observed in experiments comprise: (i) a lag of $t_{ON}\approx$ 2 seconds between the application of the stimulus and the activation of the response, (ii) a lag of $t_{peak}\approx$ 5 seconds between the application of the stimulus and the peak of the response, (iii) a maximum amplitude that ranges between 1$\%$ and 2$\%$ (measured as percentage change from the basal level) and (iv) a marked post-stimulus undershoot^47,49^. As shown in Fig. 5a, all these features are correctly captured by our model. For completeness we show in the figure a fit performed with the so called canonical HRF, a phenomenological expression widely used for fMRI data analysis. As we see the HRF obtained from our model is in good agreement with the canonical HRF.

In Fig. 5b,c we show the BOLD response obtained from the simulations together with the results from Eq. (13) (for a pulse and an oscillatory stimuli respectively). As we can see the HRF formalism can correctly reproduce the simulations result within the predicted linear range. Thus, in our model the calcium pathway generates a neurovascular coupling that behaves linearly close to the threshold of BOLD (and calcium) activation ( $\nu _{ext}\approx 2.4$ ) and for a range of more than a twofold change in neuronal activity which accounts for a about 75$\%$ of the total change in BOLD signal in our simulations.

Based on the analysis performed so far and the results shown in Fig. 4, we propose now to use the calcium activity as a predictor of the BOLD response instead of the neuronal activity. In Fig. 4h we can see that the BOLD follows a linear relation with the calcium firing rate ($f_{Ca}$), which makes it a good candidate for the HRF formalism. Thus, we will replace the function *r*(*s*, *t*) from Eq. (13) by *c*(*s*, *t*), corresponding to the calcium activity. In Fig. 5d,e) we show the results of the convolution between HRF and the time course of the calcium activity. We see that the convolution can correctly reproduce the results of the simulations for barely the entire range, except close to the threshold where the BOLD-$f_{Ca}$ relation deviates from the linearity.

In this section we have proved that the calcium activity is a good indicator of the BOLD activity and that it can be incorporated to the linear HRF formalism, exhibiting a range of validity larger than its neuronal counterpart. In addition, the fact that the HRF formalism is compatible with our model (and that our HRF is equivalent to the canonical HRF), indicates that the analysis performed in this paper for the calcium pathway of neurovascular coupling can be generalized to other models. Independently of the exact path that links the calcium activity with the vascular system, we have shown that the calcium-vascular link can be replaced by the linear HRF formalism using a canonical HRF which are experimentally tested and widely accepted. This makes the analysis presented in the following sections extensible to other alternative paths as far the linearity of the calcium-vascular relation remains valid. The linear and non-linear regimes of the coupling in our model depend mainly on the transfer function (frequency response) of the astrocytic calcium dynamics. The origin of the saturation can be traced to both the IP3 and the pure calcium dynamics from the Li-Rinzel model. In the Li-Rinzel model, the frequency of calcium-spikes is given by the concentration of IP3 and it exhibits a frequency-lock for sufficiently high concentrations of IP3 (i.e. the frequency remains unchanged for higher values of IP3). In addition, the IP3 dynamics used in our model (which depends on the glutamate concentration) exhibits a saturation in the maximum concentration of IP3 for sufficiently high values of glutamate. The glutamate and IP3 concentrations at which saturation appears depend primarily on the maximum rates of glutamate-dependent IP3 production and on the affinity of IP3-receptors in the membrane of the endoplasmic reticulum that mediate the calcium release. Finally, we notice that, although the HRF is driven by the calcium dynamics (being a single calcium-spike the minimum activation ’unit’), the characteristic times of the HRF ($t_{ON}$, $t_{peak}$) depend on the whole dynamics of the system. Thus, even when the calcium-spike frequency changes with the stimulus intensity, these changes have a relative small impact in the characteristic times of the HRF. The fact that the shape of the HRF does not change with the stimulus strength is a fundamental feature for the validity of the HRF formalism (see, for example, time-contrast separability in ref.^49^).

### BOLD undershoot and calcium dynamics

One characteristic feature of the BOLD response is the presence of a post-stimulus undershoot. The origin of this undershoot is usually attributed to three main sources: a) a slow recovery of the venule volume^19,54^; b) sustained post-stimulus metabolic rate of oxygen^42^; and c) a post-stimulus undershoot in cerebral blood flow (CBF)^54^. The first of this sources is captured by the Balloon model^19^ and is thus incorporated in our simulations. In this section we will focus on the third of this sources, namely the post-stimulus undershoot in CBF. The second source is not incorporated in the current version of our model, but it might be object of future analysis.

**Figure 6:**
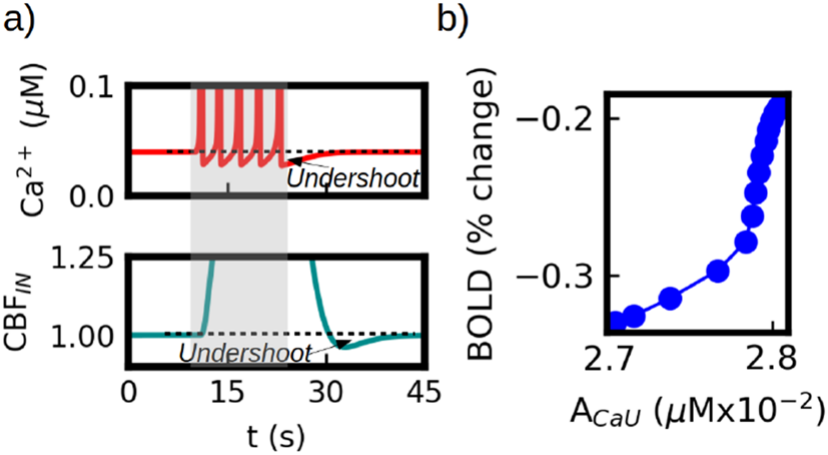
Calcium effects in BOLD post-stimulus undershoot. (**a**) Top panel: calcium time course during stimulation. After the stimulus is removed the calcium signal remains below its basal level and slowly recovers (indicated as *Undershoot* in the figure). The depressed calcium level leads to a post-stimulus undershoot in the blood flow (CBF$_{IN}$) which is shown in the bottom panel. (**b**) Evolution of the BOLD undershoot amplitude as a function of the calcium undershoot amplitude. We see that the changes in the calcium level lead to a variation of 50$\%$ in the BOLD undershoot.

In our model the level of CBF is modulated by the calcium activity. In Fig. 6a we show the time course of the calcium signal and the CBF$_{IN}$ during the application of a stimulus. We see that after each calcium-spike there is a period where the Ca$^{2+}$ level goes below its basal state. This is generated by the fast decrease of IP3 levels driven by the calcium-spike (for details see Supp. Inf. Materials and Methods). While the stimulus is applied the IP3 levels rise again and a new spike is generated. However, in the post-stimulus phase the IP3 level slowly recovers until it reaches its basal concentration. During this last period the Ca$^{2+}$ level also remains below its basal concentration giving place to a post-stimulus undershoot in the calcium signal.

This undershoot is transmitted to the CBF$_{IN}$ as shown in Fig. 6a (bottom panel). The size and duration of this undershoot depends on the intensity of the stimulus and on the phase of the calcium oscillation. In Fig. 6b we show the relation between the amplitude of the BOLD undershoot and the amplitude of the calcium undershoot (A$_{CaU}$), measured as the minimum value of the BOLD and calcium signals respectively. We see that the calcium undershoot in our simulations can account for up to 50$\%$ change of the BOLD undershoot.

### Adaptation effects

In this section we analyze the effects of neuronal adaptation in the neurovascular coupling. In our model the adaptation is described within the mean-field formulation by the variable *W* (see Eqs. 1 and 2 ), which accounts for the average adaptation of the AdEx neuronal population. We focus here only on the spike-triggered adaptation characterized by the parameter *b* (we use *a*=0 in Eq. 2). The action of adaptation in neuronal activity can be seen in the first and second panels (from the top) of Fig. 3. At the onset of the stimulus the neuronal activity exhibits a large overshoot generated by the interplay of the fast neuronal response and the slower adaptation variable *W*. Similarly, an undershoot in the neuronal activity is observed after the end of the stimulus, generated by the same mechanism. This dynamics is also captured by the glutamate concentration, which follows the variations in neuronal activity. If the characteristic time of the neuronal adaptation (given by $t_w$) is much smaller than the characteristic time of the calcium response, then the adaptation effects become negligible for the neurovascular coupling and no effects are observed in the BOLD signal. This has been the case for the simulations presented in the previous sections, where we adopted $t_w$=1s.

**Figure 7:**
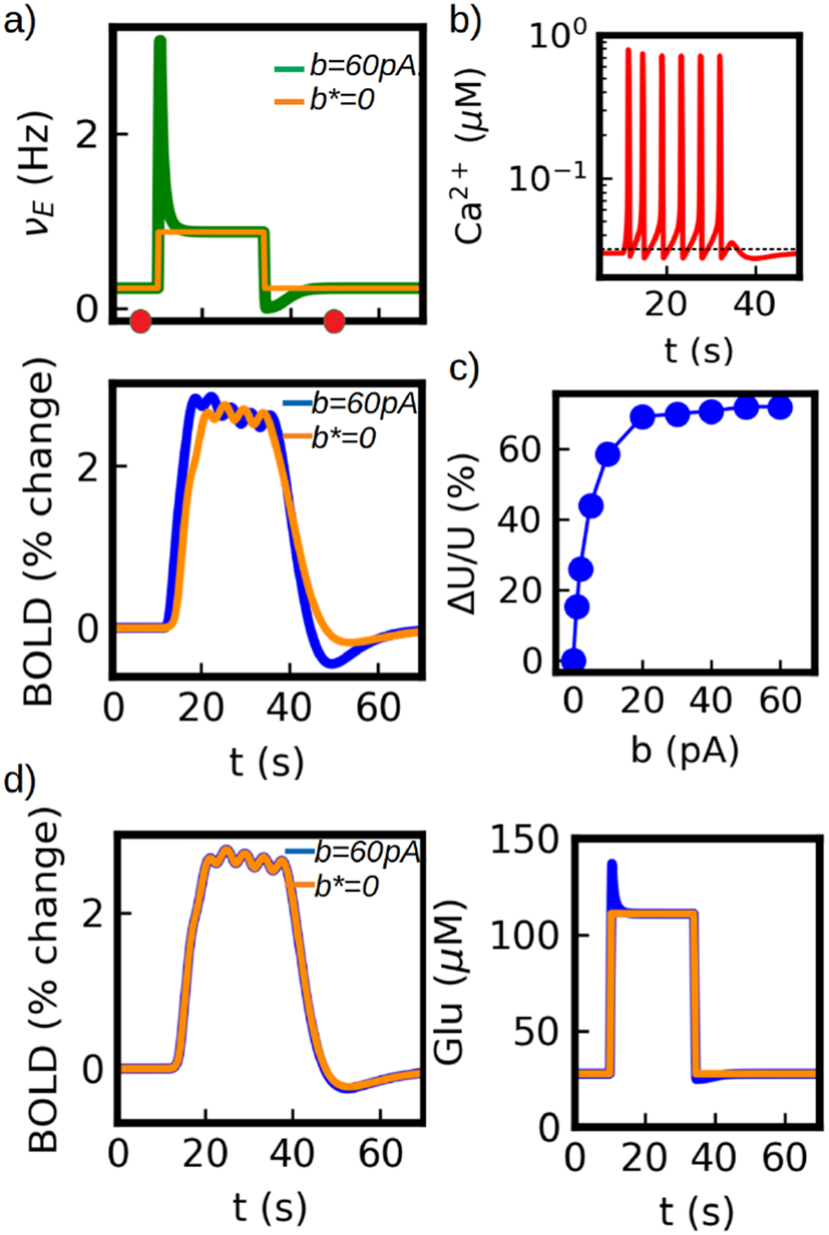
Neuronal adaptation effects in BOLD signal. (**a**) Top: neuronal activity during stimulus application with (green) and without (orange) adaptation (for a better comparison, we re-scale the asymptotic firing rate for $b=0$ to match the one of $b=60$pA, so we indicate it with $b^*$ ). Bottom: BOLD response with (blue) and without (orange) adaptation. The effects of adaptation can be observed in the higher initial response for the blue curve and in the larger post-stimulus undershoot. The simulations correspond to $t_w$=5s. (**b**) Calcium-spike adaptation. The frequency and amplitude of the calcium-spikes are modulated by the changes of glutamate concentration due to neuronal adaptation. In the figure we can see the adaptation effects occurring during the first three calcium-spikes after which a steady state is reached. In addition, a decrease in the post-stimulus calcium concentration is induced by the neuronal post-stimulus undershoot. The calcium time course shown correspond to the lapse within the red circles indicated in top panel (**a**). (**c**) Adaptation effects in the post-stimulus undershoot (PSU) of the BOLD response. The plot shows the variation in the PSU of the BOLD response as a function of the adaptation constant *b* (see Eq. 2). The variations in the undershoot are measured as $\Delta$U/U=(min(BOLD)-min(BOLD$_{Ad}$))/min(BOLD), where min() indicates the minimum value and BOLD$_{Ad}$ correspond to the BOLD response when neuronal post-stimulus undershoot is removed. In our simulations the undershoot of the neuronal activity is responsible of up to about 70$\%$ of the BOLD PSU for the highest values of *b* (with t$_W$=5s). (**d**) Simulation results for $g_r=g_{ext}$ (equal contribution of external and recurrent activity to glutamate release). We show the BOLD response (left) and glutamate concentration (right). In this case the effects of neuronal adaptation on the BOLD signal are neglectable. The comparison with previous panels ( $g_{ext}=0$), indicates that information about relative role of recurrent activity can be extracted from the post-stimulus undershoot.

The situation changes as the characteristic time of adaptation becomes comparable to the time of the calcium response. In Fig. 7 we show the results for the case $t_w$=5s. In panel (a) we show the neuronal (top) and BOLD responses (bottom) and we compare them with results having no adaptation (b*=0, see caption in Fig. 7). In this case the adaptation effects are clearly observed in the BOLD signal. At the onset of the stimulus we observe the larger BOLD response in the blue curve ($b\ne 0$) driven by the initial neuronal overshoot. Although the variation is small, we see that the response is not only larger but its maximum value is reached earlier than expected when no adaptation is considered, which is a feature observed and discussed in experiments^49^. In addition, after the stimulus, we see a large variation in the amplitude of the undershoot on the BOLD signal. These two effects can be traced to the calcium dynamics shown in panel (b). Here we can see the effects of the adaptation acting on the frequency and amplitude of the calcium-spikes at the onset of the stimulus: the initial increase in glutamate concentration generated by the neuronal overshoot leads to a higher calcium frequency and amplitude which quickly decreases and reaches a lower steady state after a few seconds. This leads to the larger initial BOLD response. On the other hand, the post-stimulus neuronal undershoot generates a decrease in the post-stimulus calcium concentration which is the origin of the larger undershoot observed in BOLD response. In panel (c) we show the variations in the BOLD undershoot as a function of the adaptation parameter *b*. We see that the post-stimulus neuronal undershoot can account for up to 70$\%$ of the BOLD undershoot for the larger values of *b* (for the details of the plot see caption). We notice that this neuronal dynamics also leads to a dependence of the PSU on the duration and strength of the stimulus. For short stimulus (compared to the *W* response) the influence of neuronal adaptation will be limited, for which a smaller neuronal-driven PSU in the BOLD signal is obtained.

Finally, we notice that the results presented so far correspond to the case $g_{ext}=0$ in Eq. (3), meaning that the only source of glutamate release is the recurrent synaptic activity (which represents the output of the local neuronal population). As we explained in previous sections, when $t_w$ is small then the results obtained for the BOLD signal are nearly independent of the choice of $g_{r/ext}$. However, when $t_w$ is increased and the adaptation effects are captured by the BOLD signal as in this section, then the relative contribution of the recurrent and incoming synaptic activity to glutamate release becomes relevant (where we assume that the incoming activity exhibits no adaptation). In Fig. 7d we show the results for the case $g_{ext}=g_r$ (i.e. equal contribution). We show in the figure the time course of the glutamate concentration (right panel), where we can see that the effects of the neuronal adaptation are drastically reduced in comparison to the case with $g_{ext}=0$. Furthermore, we can see that there is no observable variation in the BOLD signal (left panel) for the simulations with or without adaptation. In particular, the post-stimulus undershoot in the BOLD signal is no longer influenced by the neuronal adaptation. This indicates that the amplitude of the PSU depends on the relative size of the input and output (recurrent activity) of the local neuronal population. Thus, one possible interpretation is that the amplitude of the BOLD signal provides information mainly about the input to the neuronal population, while the amplitude of the PSU provides information about its output.

### Model robustness and further comparison with experimental results

In this section we analyze some important aspects related to the robustness of our model and we perform further comparison between our simulations and experimental results.

**Figure 8:**
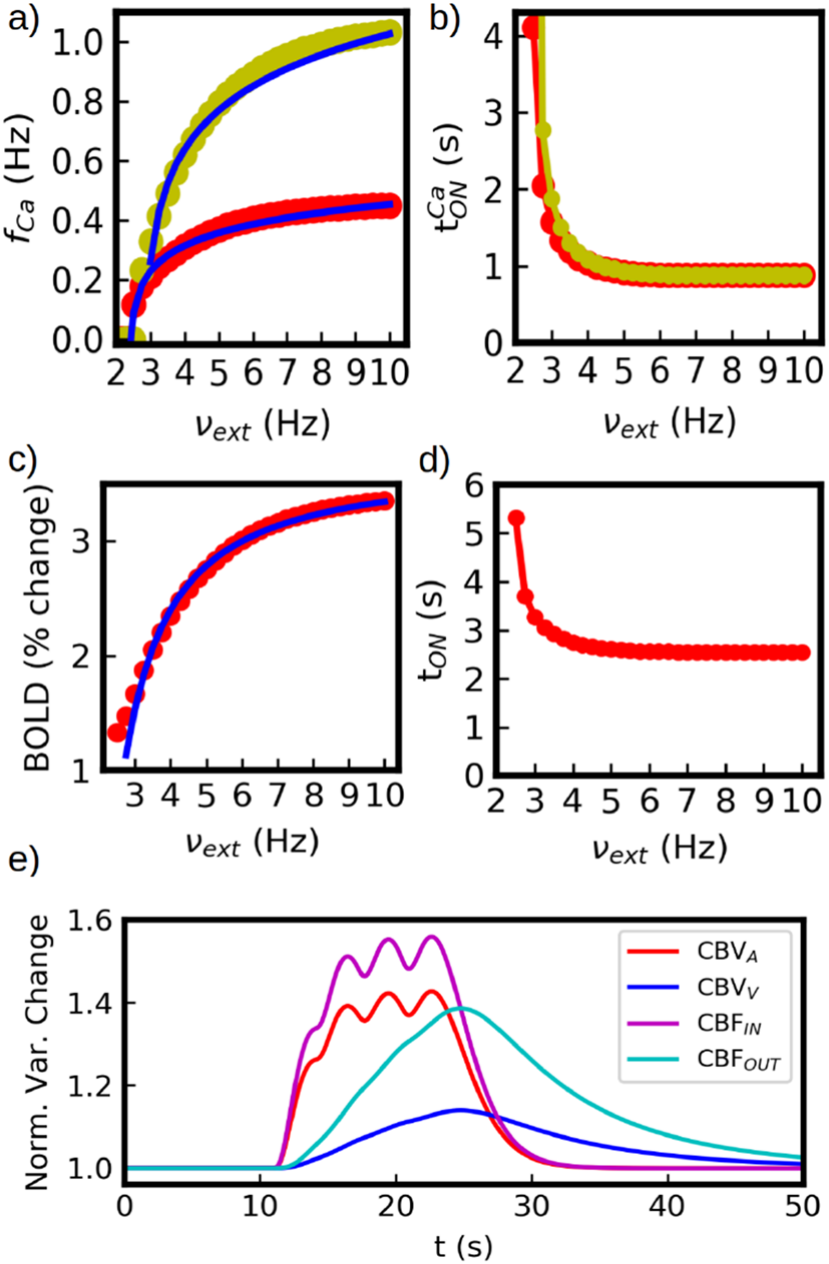
(**a**) Calcium spike frequency as a function of the external neuronal (excitatory) firing rate. We show the results for two different set of parameters. In red, the same parameters used for the rest of the paper. In green, parameters leading to a shorter calcium refractory period and higher maximum frequency rate. These results correspond to $O_2=0.2x10^3$ (mMs)$^{-1}$ (red) and $O_2=0.2x10^4$ (mMs)$^{-1}$ (green) in Eq. (5). The rest of the parameters remain unchanged (see Tables 1 and 2 in the Supp. Inf.). We see that the rate increases by a factor larger than two between the two set of parameters. For both cases we show a fit with a logarithmic function (solid blue line), as explained in Section “Calcium coding and BOLD response”. (**b**) Calcium response onset time (t$^{Ca}_{ON}$) as a function of external excitatory firing rate. We see that the time increases rapidly near the threshold for calcium activation, but then it quickly converges to a value close to 1 s. (**c**) Amplitude of the BOLD response as a function of the external excitatory rate (red). We show a fit on the response with a phenomenological expression used in experimental results^49^ (solid blue line, see main-text). (**d**) Onset time of the BOLD response. In agreement with panel (**b**) we see that the time increases rapidly close the threshold for calcium activation and then it quickly converges to a value close to 2 s. (**e**) Dynamics of the vascular response during external stimulation. We show the normalized variable change (Norm. Var. Change) of the Cerebral Blood Volume at the arteriole (CBV$_A$) and venule level (CBV$_V$), together with the incoming and outgoing blood flow (CBF$_{IN}$, CBF$_{OUT}$).

Among the different components of the model presented in this paper, one of the main novelties is the inclusion of the detailed astrocytic calcium dynamics as a key element to explain main aspects of the neurovascular coupling. In particular we have seen that the frequency-input relationship emerging from the modified Li-Rinzel model for $Ca^{2+}$ plays a key role in the comunication between the neuronal and vascular systems. Although a detailed analysis of the Li-Rinzel model has been performed before^26^ (and is out of the scope of this paper), it is important to mention a few aspects that are relevant to our current study. We start by noticing that in the Li-Rinzel model Ca-spikes are triggered via IP3 gated channels (see Eqs. 4 and 5). The deinactivation time of these channels gives place to a ’refractory-period’ for Ca-spikes which determines the maximum spiking frequency. Thus, the frequency range of calcium spikes can in principle be modulated by the deinactivation time. In Fig. 8a we show the results of the frequency-input curve for different values of the parameters from Eq. (5). In the figure, the frequency range is doubled from the original, still holding the same functional dependence (a fit with a logarithmic function is shown for each case). Although we have adapted for our main study a set of parameters previously used in the literature^26,31^, this shows that the analysis performed here remains valid for larger ranges on the frequency-input relation, which may also lead to a larger range in the BOLD response.

A second important aspect of the calcium dynamics is its response time after the application of the stimulus. It has been experimentally observed that a typical interval of approximately 1 s occurs between the astrocyte and CBF activation^55^, while another interval of approximately 1 s is expected between the neuronal and astrocytic responses^24^. The sum of these two intervals constitutes the total neuronal-to-CBF response time, which is captured in the HRF shown in previous sections. In our model, the response time of the astrocyte depends in principle on the strength of the stimulus and is not a constant, as shown in Fig. 8b. Nevertheless, we notice that this time converges quickly to a stationary value near 1 s, while it rapidly increases as it gets close to the threshold for astrocytic $Ca^{2+}$ activation. This type of non-linearities are actually expected in vascular responses for short or low amplitude stimuli^56^. The quick convergence of the activation time is of great relevance for the validity of the HRF analyisis, as it guaranties the invariance of the HRF.

To provide a better quantitative comparison between our model and actual experimental results, we analyze now the dependence of the amplitude of the BOLD signal on the strength of the stimulus. It has been shown in fMRI experiments that the amplitude of the BOLD signal can be described by the expression $f(x)=\frac{ax^p}{x^p+b}$, where *x* is the input strength (contrast) and *a*, *p*, *b* are constants^49^. In Fig. 8c we show the results of the fit of our simulations with this expression. We obtain $p=1.08$ which is in agreement with the experimental results^49^ ($a=3.69$ and $b=0.95$ are parameters that depend on the particular range of the stimulus and amplitude). This provides further validation to the BOLD signal obtained from our model. In addition we show in Fig. 8d the evolution of the onset time $t_{ON}$ of the BOLD response as a function of the stimuli. In agreement with the calcium response discussed before, it experiences a non-linearity close to the threshold for the response activation and converges rapidly to a stationary value of $\sim$2.5 s in agreement with experimental results. As mentioned before, this non-linearity is also observed in experimental measurements for short or low amplitude stimuli^56^.

Finally, for completeness we show in Fig. 8e the evolution of the vasculature volume and blood flow under the presentation of a stimulus. We show here the normalized variation of the volume at the arteriole and venule level, together with the incoming and outgoing blood flow. The amplitudes obtained for the different variables are in agreement with typical experimental results obtained via MRI and arterial spin labelling measurements^57,58^. In our simulations arterioles experience variations in volume ($CBV_A$) during functional hyperemia with changes from 20$\%$ to more than 60$\%$, while changes in venules volumes ($CBV_V$) can go up to 20$\%$. Similarly, relative changes in cerebral blood flow (*CBF*) can reach more than 70$\%$ and are related with the *CBV* via Eq. (11).

## Discussion

In this paper we have presented a new framework for modeling the neurovascular coupling. This framework is centered on calcium activity in astrocytes, that acts to link the neuronal activity to the vascular system. Starting from a relatively detailed description of the neuron-astrocyte-vascular system, we have focused on fundamental aspects of the fMRI phenomenology such as the different experimental designs, the Hemodynamic Response Function (HRF), the linearity in the coupling, the post-stimulus undershoot and the effects of neuronal adaptation. We have shown that calcium signaling in astrocytes may play a relevant role in all of these features. Previous papers with models of the neuron-astrocyte-vascular interaction have been proposed^21–24^. Some of these models proposed a phenomenological approach to describe the role of astrocytes^24^ or studied limited aspects of the neurovascular coupling^21–23^. To the best of our knowledge, the present work is the first one that accounts for the above experimental observations on fMRI.

We have shown that the calcium-driven HRF generated by our model is equivalent to the one observed experimentally and we provided a comparison with the canonical HRF.

We have seen that calcium signaling can explain the linear and non-linear features of the neurovascular coupling. The non-linear features involve the existence of a threshold of neuronal activity for vascular activation and saturation in the BOLD signal for sufficiently high neuronal activity. In parallel our simulations predict a dynamical range where the coupling is linear. The linear coupling comprises around 75$\%$ of the total variation in the BOLD signal explored in our simulations and corresponds to the typical range of values seen in experiments (variations in the BOLD signal between 1 and 3 $\%$). We have shown that within this range we can reproduce our simulations with the HRF linear formalism, which is widely used for data-analysis of fMRI experiments. In addition our model predicts that the BOLD signal is linearly coupled with the calcium activity, for which we have tested the HRF formalism with the calcium activity (instead of neuronal) which provides better results.

The way that calcium signal codifies and transmits information from the neuronal to the vascular system was studied. We have found that, in our model, the coding is performed mainly via frequency modulation of calcium-spikes with a small contribution of amplitude modulation (see Supp. Inf. for a discussion of the BOLD response to neuronal activity at different frequencies).

Other important aspect studied in the paper is the role of calcium signal on the BOLD post-stimulus undershoot (PSU). We have explored the contributions of neuronal and calcium activity to the PSU. We have shown that an undershoot in calcium concentration, which emerges from the same calcium dynamics, has a relevant contribution to the PSU. The recovery of the basal calcium concentration occurs faster than the the recovery of the BOLD PSU, which indicates that calcium might have a stronger contribution on the early phase and on the amplitude of the PSU. Such a calcium undershoot can be observed experimentally^16,59–62^ and recently its effect on the arteriole tone has been described^63^ , but, to the best of our knowledge, has not been studied so far in relation to functional hyperemia and the BOLD signal.

In addition, we have shown that the inclusion of neuronal adaptation generates a post-stimulus undershoot in the neuronal activity which is captured by the calcium signal (coded in terms of calcium-spike frequency and amplitude adaptation) and transmitted to the vascular PSU. The contributions of PSU described here operate on the BOLD via the CBF, i.e. they lead to an undershoot in the CBF that is then captured by the BOLD. The existence of such neuronal-CBF-BOLD undershoot can also be observed in experimental work^54,64^. We note that the undershoot of neuronal activity is independent of the astrocyte-calcium pathway and its impact on the BOLD might be taken into account for direct neuron-vascular interaction. Furthermore, it is believed that the direct neuron-vascular pathway also involves calcium activity as a mediator^6,8^, for which the results obtained from our model might be generalized for alternative neurovascular pathways. The impact of neuronal adaptation on BOLD signal has proven to be of importance in fMRI experiments and it has led to the development of fMRI-adaptation, a technique that makes use of neuronal adaptation and neuronal specificity. In this context the adoption of the AdEx formalism constitutes an important step towards an accurate description of adaptation effects in the neurovascular coupling, representing a relevant improvement with respect to standard neuronal-mass and field models used for fMRI analysis^65^.

As mentioned in the introduction to this paper, there is still a debate around the role of astrocytes in neurovascular coupling. In this work we have provided a complete new analysis on how astrocytes can be acting in the coupling. Furthermore, we have provided a series of results linking calcium dynamics to the BOLD signal that can be experimentally studied and tested (such as the frequency-coding mode, the linearity of this response, the role of calcium in the post-stimulus undershoot, etc.). We hope that our work can contribute to this debate and provide tools for future experimental analysis to elucidate this very relevant issue.

Possible extensions of our study may involve the description of BOLD imaging to a larger scale (multiple voxels or brain regions) and the analysis of resting state fMRI. There is evidence that astrocytes may also mediate vasoconstriction, which may depend on the metabolic state of cerebral tissue^30^. The role of astrocytes in vasoconstriction can also be incorporated in our model. Indeed, in the current version of the model astrocytes contribute to the basal tone of blood vessels via the maintenance of *cAMP* levels in smooth cells. In the Supp. Inf. we show how astrocyte-mediated vasoconstriction can be incorporated to our model and its potential role in vascular homeostasis (Supplementry information Fig. S3). In addition, it has been recently suggested that astrocytes may play a key role in the amplification of functional hyperemia to sustained stimulation, which could be also be part of future studies within our model^66^. On the other hand, inhibitory neurons may also be involved in Functional Hyperemia^29^, for example via the release of nitric oxide (which is a known vasodilator) or even via interaction with astrocytic end-feet as has recently been suggested^13^. All these represent interesting paths for future elaborations of our model. The development of realistic models of the neurovascular coupling is currently of great relevance for the analysis of clinical data^14,67^. Furthermore, the mean-field formalism adopted here provides tools to estimate multiple brain signals such as LFP and MEG^68^. This will give the chance to analyze multimodal experiments, where BOLD imaging is combined with electrical measurements, which is a topic of great interest^14^. Furthermore, our framework can be combined with different neuronal models and has the potential of being incorporated in whole brain simulators such as The Virtual Brain^44,69^.

In addition, since astrocytes play a role in several neurodegenerative diseases^70–72^, our modelling framework may be investigated to predict how astrocytic dysfunction in brain disorders can result in detectable changes in neurovascular coupling and BOLD signal. For example, mutations in the *PLA2G6* gene, associated to Parkinson’s disease^73^, can lead to a disrupt in the release of Arachidonic Acid from membrane phospholipids and a reduction in calcium responses^72,74^, which could be captured by our model (see Supp. Inf. for an example of astrocytic dysfunction, Fig. S4).

Finally, our model may be used for inferring neuronal activity from the BOLD signal, by reversing the equations (see Supp. Inf. for an example). In principle, this could yield to estimates of the global glutamatergic activity, and thus estimates of the population activity of excitatory neurons. Such estimates should be compared to simultaneous measurements of population activity and BOLD signal. As BOLD-fMRI is one of the leading non-invasive research tools in neuroscience, finding new and better methods to infer neuronal or calcium activity constitutes a promising direction for future work. Such methods can be of great relevance for studies of human brain networks in health and disease.

## Supplementary Information

Supplementary Information.

## Data Availability

The code used to generate the data analysed during the current study is available in the Zenodo repository, 10.5281/zenodo.7801673.
