# Supplementary material for: Modeling the relationship between neuronal activity and the BOLD signal: contributions from astrocyte calcium dynamics
Source: Sci Rep. 2023 Apr 20;13:6451. doi: 10.1038/s41598-023-32618-0 (PMC10119111; doi:10.1038/s41598-023-32618-0)
Supplement: Supplementary file 1 — Supplementary Information. [file 41598_2023_32618_MOESM1_ESM.pdf]

Supplementary Information

1. Materials and Methods

1.1. Neuronal model

In our paper we consider a network made of Adaptive Exponential Integrate and Fire neurons (AdEx) [1]. The equations for the AdEx model are given by:

$$\begin{aligned} c_m \frac{dv}{dt} &= g_L(E_L - v) + \Delta e^{v - v_{th}} - w + I_{syn} \\ \frac{dw}{dt} &= -\frac{w}{\tau_w} + b\delta(t - t_{sp}) + a(v - E_L) \end{aligned} \quad (1)$$

where $c_m = 200$ pF is the membrane capacity, v is the voltage of the neuron, and whenever v>v$_t h = -50$ mV at time tsp(k), v is reset to the resting voltage $v_{rest} = -65$ mV and fixed to that value for a refractory time $T_{ref} = 5$ ms. The leak conductance is $g_L = 10$ns and the leakage reversal potential is E_L=−70 mV. The exponential term has a different strength for excitatory and inhibitory cells $\Delta = 2$ mV (= 0.5 mV) for excitatory (inhibitory) cells. We consider inhibitory neurons with no adaptation ($a = b = 0$) and for excitatory neurons we take $b = 60$pA and $a = 0$. For the recovery time of adaptation we take $t_w = 1$s except otherwise indicated. The synaptic current I_{syn} received by a neuron i is the result of the spiking activity of all presynaptic neurons j ∈ pre(i) of neuron i. This current can be decomposed in the input received from excitatory E and inhibitory I presynaptic spikes $I_{syn} = (E_e - v)G^e_{syn} + (E_i - v)G^i_{syn}$, where Ee = 0 (Ei = −80 mV) is the excitatory (inhibitory) reversal potential. We consider voltage dependent conductances. We model the conductances G^e_{syn} as a decaying exponential function that takes kicks of amount Q_E at each presynaptic spike,

$$G^e_{syn} = Q_e \sum_{excit.pre} \Theta(t - t_{sp}) e^{-\frac{t - t_{sp}}{\tau_e}} \quad (2)$$

where the sum goes through all presynaptic excitatory spikes, Θ is the heaviside function, $\tau_e = \tau_i = 5$ ms is the decay timescale of excitatory and inhibitory synapses, and $Q_e = 1.5$ nS ($Q_i = 5$ nS) the excitatory (inhibitory) quantal conductance (i.e. the change in conductance generated by a single spike). We use the same equation with $e \rightarrow i$ for inhibitory neurons.

The use of AdEx neurons provides a realistic description of neuornal activity and allows us to explore adaptation effects over the hemodynamic response. In addition, the spatial scale of the hemodynamic response suggests that vascular signals are affected by the population activity rather than by single neuronal dynamics. The strong correlation of the BOLD response with signals such as LFP also points in this direction [2]. Furthermore, each astrocyte in the brain interacts with thousands to millions of synapses [3, 4], suggesting that these cells are capable of sensing population activity. Thus, the choice of a mean-field formulation emerges naturally in this context.

1.2. Astrocytic Calcium Dynamics

To describe the astrocytic calcium dynamics we adopt a recent version of the Li-Rinzel model by De Pittá et al. [5, 6]. This model describes the activation of metabotropic glutamate receptors in the astrocyte, the production and degradation of inositol trisphosphate (IP3) and the flux of Ca^{2+} between the cytosol and the ER of the cell. The fraction of activated glutamate receptors (γ_A) is given by:

$$\partial_t \gamma_A = -\frac{\gamma_A}{\tau_A} + O_M(1 - \chi) Glu(1 - \gamma_A) \quad (3)$$

where $(1 - \chi)$ is the fraction of glutamate that spilled out of the synaptic cleft, τ_A is the characteristic receptor deactivation (unbinding) time constant, O_M is the binding rate and Glu is the glutamate concentration in the synaptic cleft. The values of the parameters are shown in Supplementary Table 2.

The IP3 concentration results from the Ca^{2+}-modulated interplay of phospholipase Cβ- and Cδ-mediated production and degradation by IP3 3-kinase (3K) and inositol polyphosphatase 5-phosphatase and evolves according to [6]:

$$\partial_t IP3 = J_\beta(\gamma_A) + J_\delta(Ca^{2+}, IP3) - J_{3k}(Ca^{2+}, IP3) - J_{5P}(IP3) \quad (4)$$

where

$$
\begin{aligned}
J_\beta &= O_\beta \gamma_A \\
J_\delta &= O_\delta \frac{\kappa_\delta}{\kappa_\delta + IP3} H(Ca^{2+2}, K_\delta) \\
J_{3K} &= O_{3K} H(Ca^{2+4}, K_D) H(IP3, K_3) \\
J_{5P} &= \Gamma_{5P} IP3
\end{aligned}
\tag{5}
$$

Here $H(x^n, K)$ denotes the Hill function $x^n/(x^n+K^n)$. Cytosolic calcium concentration and IP3 gating are described according to the Li-Rinzel model [7]:

$$
\begin{aligned}
\partial_t C^{2+} &= J_C(Ca^{2+}, h, IP3) + J_L(Ca^{2+}) - J_P(Ca^{2+}) \\
\partial_t h &= \frac{h_\infty(Ca^{2+4}, IP3) - h}{\tau_h(Ca^{2+4}, IP3))}
\end{aligned}
\tag{6}
$$

where J_C , J_L , and J_P , respectively, denote the IP3-mediated Ca^{2+} -induced Ca^{2+} -release from the ER (J_C), the Ca^{2+} leak from the ER (J_L), and the Ca^{2+} uptake from the cytosol back to the ER by serca-ER Ca^{2+} /ATPase pumps (J_P) [141]. These terms, together with the IP3R deinactivation time constant (τ_h) and steady-state probability (h_∞), are given by

$$
\begin{aligned}
J_C &= \Gamma_C m_\infty^3 h^3 (C_T - (1 - \rho_A) Ca^{2+}) \\
m_\infty &= H(IP3, d_1) H(Ca^{2+}, d_5) \\
J_L &= \Gamma_L (C_T - (1 - \rho_A) C) \\
J_P &= O_P H(Ca^{2+}, K_P) \\
h_\infty &= d_2 \frac{IP3 + d_1}{d_2(IP3 + d1) + O_2(IP3 + d_3) Ca^{2+}} \\
\tau_h &= \frac{IP3 + d_3}{\Gamma_2(IP3 + d1) + O_2(IP3 + d_3) Ca^{2+}}
\end{aligned}
\tag{7}
$$

The values of the parameters are given in Supplementary Table 2.

In this model the information about the input (i.e. glutamate concentration) is codified in terms of both the Amplitude and Frequency of calcium oscillations [5], as described in the main text. The relative relevance and efficiency of each type of coding depends on the set of parameters used and, in particular, in the duty-ratio of the calcium oscillations (i.e. the ratio between the width of the calcium pulse (spike) and the period) [5, 8]. Experimental and theoretical data suggests that the dominating coding mode of information is via frequency modulation [5, 9, 10, 11]. For this reason, we considered in this work relative low duty-ratio (<0.5) calcium oscillations which are more likely associated with efficient frequency coding [8]. On the other hand, high duty-ratio (smooth) calcium oscillations have been associated with the coordination of information between multiple astrocytes via intercellular signaling [5, 12]. Further analysis on the relative relevance of the coding modes can be an interesting topic for future studies, with the possibility that astrocytes may change the relation between coding modes depending on brain states, which could be of relevance for the analysis of resting-state fMRI.

Finally, we notice that the post-stimulus undershoot proper to the calcium dynamics studied in the main text depends on the IP3 oscillations around the basal state, which is a particular feature of the modifications introduced by De Pittá et al [5] to the original Li-Renzel model [7]. On the contrary, the post-stimulus undershoot driven by neuronal adaptation effects is the result of a decrease in the glutamate level (which in turns generate a decrease in the IP3 levels), and it is not specific to this particular model.

1.3. The Balloon model

To describe the generation of the BOLD signal we adopt the Balloon model [13, 14]. This model describes the change in dexohemoglobin concentration (which originates the BOLD signal) generated by an increase in CBF. The equations of the model are given by:

$$
\begin{aligned}
\partial_t CBV_V &= \frac{(CBF_{IN} - CBV_V^{1/\alpha_V})}{\tau_{vf}(1 + a_{fout} CBV_V^{-0.5}/\tau_{vf})} \\
CBF_{OUT} &= CBV^{1/\alpha_V} + a_{fout} CBV^{-0.5} \partial_t CBV_V \\
Extr &= 1 - (1 - Extr_o)^{1/CBF_{IN}} \\
\partial_t dHB &= (CBF_{IN} \frac{Extr}{Extr_o} - CBF_{OUT} \frac{dHB}{CBV_V})/\tau_{vf}
\end{aligned} \tag{8}
$$

where CBV_V is the venous balloon volume, CBF_{OUT} is the flow of blood leaving the balloon, $Extr$ is the oxygen extraction rate, dHB is the dexohemoglobin concentration. and τ_{vf} is the mean transit time through the venous compartment at rest.

1.4. Calcium-neurovascular pathway

We present here the simulations of the entire pathway for neurovascular coupling in our model. We show the results in Supplementary Fig. 1 and 2. In Supplementary Fig. 1 we show the main variables of the neuronal and vascular systems together with the calcium activity. In Supplementary Fig.2 we show the the variables related with the arachidonic acid cascade and the cAMP dynamics.

In this paper we have focused on the excitatory synaptic activity as the main driver of the BOLD response. We notice that activity from inhibitory neurons has also been suggested as a driver for the BOLD response. It has been usually thought that excitatory synapses are the main drivers of the hemodynamic response as they represent the larger portion of energy consumption [15]. The inclusion of inhibitory neurons as a source of hemodynamics response in our model would be an interesting point for future studies. Nevertheless, we notice that excitatory and inhibitory activity are strongly correlated during stimulation [15]. In addition, the coupling with inhibitory neurons is believed to be carried either through astrocyte activation (similar to the description of our model) or by direct Nitric Oxide release which is also driven by intracellular calcium activity [16, 17]. Thus, we believe that the inclusion of inhibitory neurons as drivers of the hemodynamic response wouldn't affect the main results of the current study.

2. BOLD response to neuronal activity at different frequencies

It is known that the BOLD signal exhibits a stronger correlation with Local Field Potentials (LFP) than with multi-unit neuronal activity (MUA) [2]. LFP is a signal related to the synaptic activity at the population level while the MUA reflects the activity of a small group of neurons (in the order of the ten(s)). Within the frequency range of LFP (0-100Hz) the correlation with BOLD has shown to be stronger for the gamma band (40-100Hz) [2, 18]. The preference for the gamma frequency has been associated to its stimulus dependent response, while frequencies within the beta band (18-30Hz) have shown to be stimulus-independent and may reflect the contribution of a stimulus-independent neuromodulatory pathway [18, 19]. In addition, the alpha band (8-12Hz) has been seen to be anti-correlated with the BOLD signal (for constant total LFP spectrum power), which is attributed to the shift of the spectrum towards higher frequencies during the application of a stimulus [18]. We first notice that our model reflects the same high correlation between the BOLD signal and the synaptic activity that is contained within the LFP. The neuronal activity in our model is described via a mean-field model which, by construction, describes the activity at the population level and not at the MUA level. In addition, the main source of LFP is known to be synaptic activity (rather than spiking), which in our model is represented by glutamate release and is the driving signal for the calcium dynamics in astrocytes and the consequent BOLD response. Thus, in our model the BOLD response reflects the synaptic activity at the population level in equivalence to the LFP. Second, in our model the BOLD is stimulus-dependent for which a higher correlation with the stimulus-dependent LFP band (gamma) would also be expected. Third, the shift of the spectrum toward higher frequencies is also implicit in the mean-field model via the variation of the mean firing rate during stimulation. Thus, our model is in qualitatively agreement with the experimental observation regarding the dependence of the BOLD with the LFP signal and its frequency components.

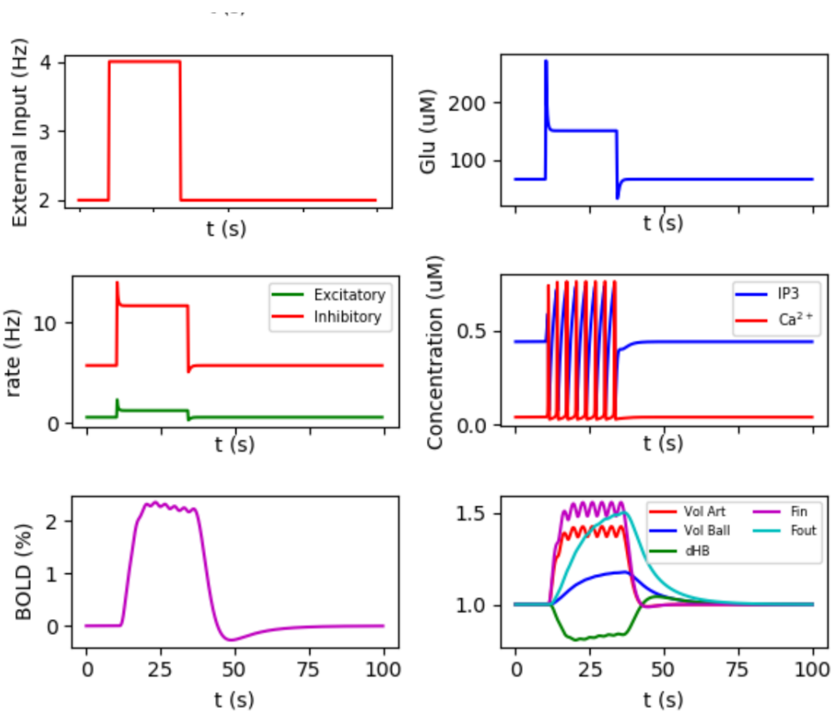

Supplementary Figure 1: In this plot we show the main variables of the neuronal and vascular systems together with the calcium activity for a pulse of ν_{ext} = 4Hz and a duration of 24s.

3. Vascular homeostasis and role of astrocyte-mediated vasoconstriction

Experimental evidence suggests that calcium activity in astrocytes can also mediate in vasoconstriction. In particular, it was observed that the dilation/constriction effect may depend on the local metabolic state of the tissue [16, 20]. It was shown that at low oxygen concentrations, astrocytic activity can lead to vasodilation, while at high oxygen concentrations it leads to vasoconstriction. One explanation proposed for this observation is that at low oxygen levels there is an increase in the production and release of lactate from astrocytes which inhibits prostaglandin transporters (PGT) and enhance the accumulation of PGE2 in the extracellular medium [16, 20]. In this section we show how this effect can be incorporated in our model. Furthermore, we show that the dilation/constriction dependence on the metabolic state works as a feedback control to regulate the oxygen concentration via variations in the CBV (cerebral blood volume) and CBF. To incorporate the role of the metabolic state in our model we notice that the action of the oxygen concentration in the prostaglandin transporters can be described in terms of the relaxation time τ_{PG} which gives a proxy of the characteristic relaxation time of PGE2 concentration.We will write $\tau_{PG} = \tau^0_{PG} + \tau_{PG}([O_2])$, where $\tau_{PG}([O_2])$ is a function that depends on the oxygen concentration and τ^0_{PG} is a constant that accounts for oxygen independent relaxation of PGE2. We will write $\tau_{PG}([O_2])$ as a Hill function:

$$\tau^{-1}_{PG}([O_2]) = \frac{[O_2]^n}{K_{O2} + [O_2]^n} \tag{9}$$

where K_{O2} is a constant and n is the Hill exponent. For simplicity we will assume that $\tau_{PG}([O_2])$ is the main contributor to the relaxation time (i.e. $\tau_{PG}([O_2]) >> \tau^0_{PG}$) and that $\tau^{-1}_{PG} \approx \frac{[O_2]^n}{K_{O2}}$. The Hill exponent n gives a measures of the strength of the oxygen dependence. We will take $n = 2$ for our simulations. To estimate the oxygen concentration in the tissue we will follow the hypothesis of a perfectly efficient O_2 metabolism assumed within the Balloon model [13, 14] (i.e. all oxygen extracted from the capillaries is metabolized). Under this hypothesis the oxygen concentration can be taken equal to the cerebral rate of oxygen consumption ($CMRO_2$), which has been observed to follow a linear relation with CBF [14]. Thus, the variation of oxygen concentration with respect to a baseline value (set at unity) can be written as $[O_2] = 1 + a_{O2}(CBF_{IN} - 1)$, where the slope $a_{O2} = 0.5$.

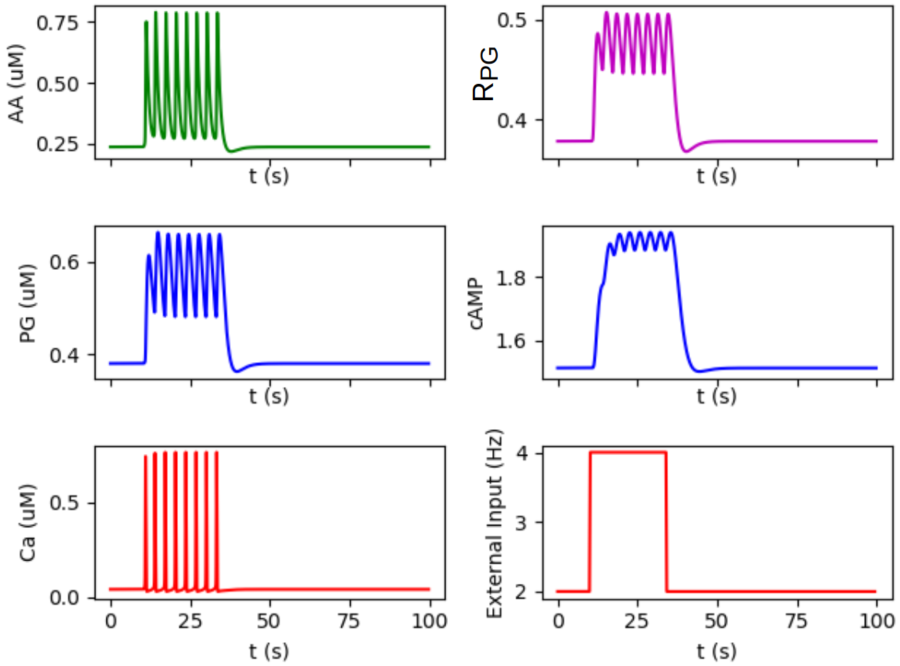

Supplementary Figure 2: In this plot we show the variables related with the arachidonic acid cascade and the cAMP dynamics for a pulse of ν_{ext} = 4Hz and a duration of 24s.

To illustrate the role of this mechanism in our model we analyze the response of the system under a sudden variation in the incoming blood flow CBF_{IN} (for a constant neuronal activity, absence of stimulus). This may represent a transient variation in the flow under normal conditions or an obstruction in the nearby vessel. In Supplementary Fig. 3 we show the results of our simulations for both a decrease and increase in CBF_{IN}. We see from our simulations that the initial decrease in the flow leads to a reduction on the oxygen concentration which in turns increases the concentration of PGE2. The increase in PGE2 acts as a regulatory mechanism generating a dilation in the vessel and an increase in the flow which increases the oxygen concentration. We see that for this simulations the regulatory mechanism provided by the PGE2 leads to a recovery of about 50% in the tissue level of $[O_2]$. An analogous situation occurs under a sudden increase in the flow, with the reduction in the concentration of PGE2 and a constriction of the nearby vessels.

4. Astrocyte dysfunction and neurovascular coupling

In this section we present an example of a dysfunction in astrocytes in our model that has repercussions in the neurovascular coupling. In Supplementary Fig. 4 we present the calcium and BOLD response when the maximum rate of calcium release from the ER towards the cytosol (Γ_C) is altered (see Eq.7). We see how this alteration generates first a reduction in the frequency of the calcium spikes and, as the alteration is increased, the spiking dynamics is completely suppressed. The alteration is also observed in the BOLD response (panel (b)), where the amplitude of the signal is reduced. In panel (c) we show the power spectrum of the BOLD response. The peak in the spectrum corresponding to the frequency of calcium spikes can be detected in the BOLD signal. This peak exhibits a shift toward lower frequencies for the altered astrocyte (middle panel) and the two first harmonics can also be detected.

5. Neuronal activity estimation from the BOLD signal

The final goal of fMRI imaging is to obtain an estimate of the underlying neuronal activity. In this context realistic models of the neurovascular coupling represent a valuable tool. The model presented in this paper provides several

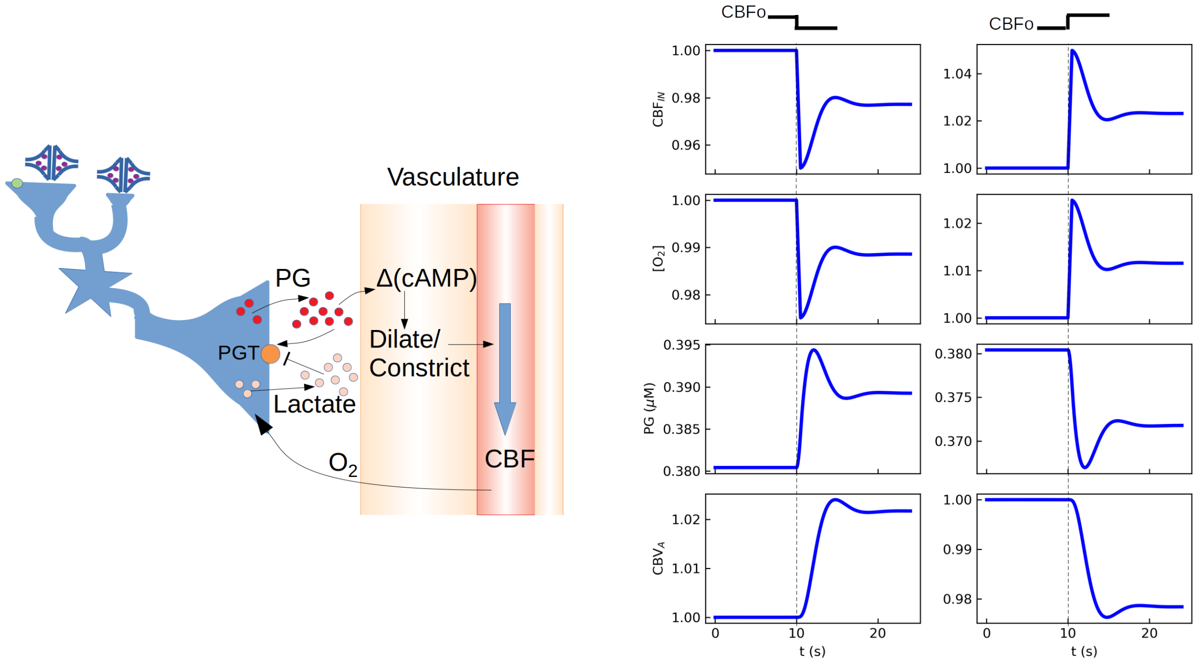

Supplementary Figure 3: Putative role of astrocytes in homeostasis and vasoconstriction. Left: Diagram of the dilation/constriction mechanism. Oxygen concentration regulates the production and release of lactate from the astrocyte. The released lactate can inhibit prostaglandin transporters (PGT), which leads to a variation of PG and cAMP concentrations. The relative variations of cAMP can induce either a dilation or constriction of the arteriole which modulates the CBF and in turn the oxygen concentration. Right: simulation's results for a sudden reduction (left) and increase (right) of the incoming blood flow (illustrated as CBF_o at the top of the plot). We see that for these simulations the regulatory mechanism provided by the interplay between oxygen concentration and PGE2 leads to a recovery of about 50% in the tissue level of $[O_2]$.

paths to perform this task. The most direct method consists in a reversion of the neurovascular pathway via the spectral analysis of the BOLD signal. As seen in Supplementary Fig. 4.c, the frequency of calcium spikes can be detected in the spectrum of the BOLD signal (peak at 0.4Hz, left panel). Then, the excitatory neuronal activity can be directly obtained via the $f_{Ca} - \nu_e$ relation (Fig.4.d, main text). In the case of Supplementary Fig. 4 (left panels a,b,c), the peak at 0.4Hz in the BOLD spectrum corresponds to $\nu_e = 2Hz$ which in turns correspond to $nu_{ext} = 6Hz$ (see Fig.4.a, main text). The amplitude of the BOLD signal can also provide a similar estimation, although this is less direct and can be affected by different elements in the coupling pathway (i.e. variations in arachidonic acid response or even changes in the basal state). Further information about the neuronal activity can be obtained from the post-stimulus undershoot (PSU) in the BOLD response. As shown in the main text, the size of the PSU depends on the relative contribution to glutamate release of the recurrent (ν_e) and incoming (ν_{ext}) synaptic activity. In our model the contribution of ν_e and ν_{ext} is weighted by the proportionality constants $g_{r/ext}$ which we use as free parameters in the main text. However, for analyzing real data, $g_{r/ext}$ are given by the proportion of recurrent and incoming synapses in the network, which is a known value for some brain regions (for example 70/30% in the visual cortex). Thus, in this case ν_e and ν_{ext} can be estimated from the data. Furthermore, the mean-field model provides straightforward ways of calculating other brain signals such as Local Field Potentials, for which analysis of multimodal data can also be developed. This last is indeed a topic of great interest [21] which opens exciting paths of research for the future.

References

[1] R. Brette, W. Gerstner, Adaptive exponential integrate-and-fire model as an effective description of neuronal activity, Journal of neurophysiology 94 (2005) 3637–3642.

[2] N. K. Logothetis, J. Pauls, M. Augath, T. Trinath, A. Oeltermann, Neurophysiological investigation of the basis of the fmri signal, nature 412 (2001) 150–157.

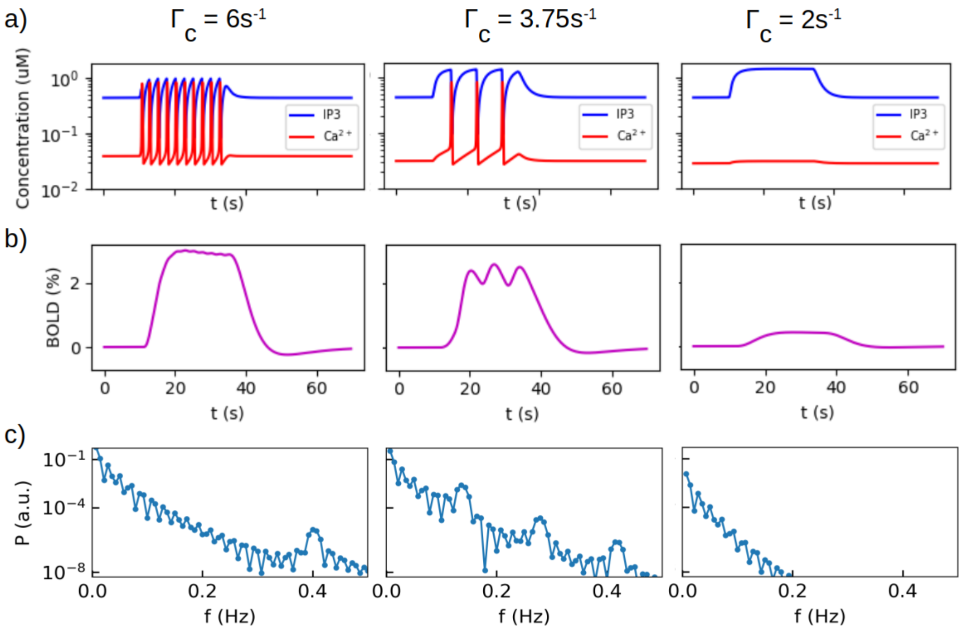

Supplementary Figure 4: Astrocyte dysfunction and neurovascular coupling. We show simulations for three different values of maximum rate of calcium release from the ER towards the cytosol Γ_C (see Eq.7). a-b) Calcium dynamics and BOLD response for a simulations of $\nu_{ext} = 6Hz$ applied for 24s. c) Power spectrum of the BOLD signal. We see that the alteration in the astrocyte causes a reduction in the frequency of calcium spikes which induces a reduce response in the BOLD signal. In addition, the variation in frequency can detected in the power spectrum where the peak corresponding to the calcium dynamics exhibits a shift toward lower frequencies, from 0.4Hz in the left panel to ~ 0.15Hz in the middle panel. The first two harmonics can also be detected in the later case. To compute the power spectrum the stimulation time was extended to 48s in order to obtain a significant number of spikes.

[3] M. M. Halassa, T. Fellin, H. Takano, J.-H. Dong, P. G. Haydon, Synaptic islands defined by the territory of a single astrocyte, Journal of Neuroscience 27 (2007) 6473–6477.
[4] N. A. Oberheim, T. Takano, X. Han, W. He, J. H. Lin, F. Wang, Q. Xu, J. D. Wyatt, W. Pilcher, J. G. Ojemann, et al., Uniquely hominid features of adult human astrocytes, Journal of Neuroscience 29 (2009) 3276–3287.
[5] M. De Pittà, M. Goldberg, V. Volman, H. Berry, E. Ben-Jacob, Glutamate regulation of calcium and ip 3 oscillating and pulsating dynamics in astrocytes, Journal of biological physics 35 (2009) 383–411.
[6] M. De Pittà, N. Brunel, Modulation of synaptic plasticity by glutamatergic gliotransmission: A modeling study., Neural plasticity (2016).
[7] Y.-X. Li, J. Rinzel, Equations for insp3 receptor-mediated [ca2+] i oscillations derived from a detailed kinetic model: a hodgkin-huxley like formalism, Journal of theoretical Biology 166 (1994) 461–473.
[8] C. Salazar, A. Z. Politi, T. Höfer, Decoding of calcium oscillations by phosphorylation cycles: analytic results, Biophysical journal 94 (2008) 1203–1215.
[9] V. Parpura, Glutamate-mediated bi-directional signaling between neurons and astrocytes, in: Glial Neuronal Signaling, Springer, 2004, pp. 365–395.
[10] L. Pasti, A. Volterra, T. Pozzan, G. Carmignoto, Intracellular calcium oscillations in astrocytes: a highly plastic, bidirectional form of communication between neurons and astrocytes in situ, Journal of Neuroscience 17 (1997) 7817–7830.
[11] X. Gu, W. Chen, N. D. Volkow, A. P. Koretsky, C. Du, Y. Pan, Synchronized astrocytic ca2+ responses in neurovascular coupling during somatosensory stimulation and for the resting state, Cell reports 23 (2018) 3878–3890.
[12] M. De Pitta, V. Volman, H. Levine, E. Ben-Jacob, Multimodal encoding in a simplified model of intracellular calcium signaling, Cognitive processing 10 (2009) 55.
[13] R. B. Buxton, E. C. Wong, L. R. Frank, Dynamics of blood flow and oxygenation changes during brain activation: the balloon model, Magnetic resonance in medicine 39 (1998) 855–864.
[14] R. B. Buxton, K. Uludağ, D. J. Dubowitz, T. T. Liu, Modeling the hemodynamic response to brain activation, Neuroimage 23 (2004) S220–S233.
[15] R. B. Buxton, The thermodynamics of thinking: connections between neural activity, energy metabolism and blood flow, Phil. Trans. R. Soc. B 376 (2020).
[16] D. Attwell, A. M. Buchan, S. Charpak, M. Lauritzen, B. A. MacVicar, E. A. Newman, Glial and neuronal control of brain blood flow, Nature 468 (2010) 232–243.
[17] M. Lauritzen, Reading vascular changes in brain imaging: is dendritic calcium the key?, Nature Reviews Neuroscience 6 (2005) 77–85.

	AdEx Mean Field Model	
N_E	#excit. neurons	8000
N_I	#inhibit. neurons	2000
p	Connection probability	5%
T	Neuronal characteristic time	5ms
τ_W	Adaptation time constant	1s
b_E	Adaptation spike-triggered constant (excit. neurons)	60pA
b_I	Adaptation spike-triggered constant (inhibit. neurons)	0
a	Adaptation sub-threshold constant (excit/inhibit neurons)	0
E_L	Leakage reverse potential (excit/inhibit neurons)	-70mV
g_r^*	Constant of glutamate release by recurrent activity	4.2x10^{-3}mM/S
	**Astrocyte**	
τ_{AA}	Relaxation time AA	1s
O_{AA}	Max. rate of AA production	5x10^{-3}mMs^{-1}
K_{AA}	Michaelis constant AA	0.8e-3x10^{-3}mM
τ_{PG}	Relaxation time PGE2	1s
O_{PG}	Max. rate of PG production	2x10^{-3}mMs^{-1}
K_{PG}	Michaelis constant PGE2	1x10^{-3}mM
	**Vascular System**	
τ_R	Relaxation time PGE2 receptor	0.8s
O_R	PGE2 binding rate	0.2x10^4(mMs)$^{-1}$
τ_{cAMP}	Relaxation time $cAMP$	2s
O_{cAMP}	Max. rate of $cAMP$ production	2x10^{-3}mMs^{-1}
D_A	Max. Arteriole dilation	5
K_{VA}	Arteriole-$cAMP$ half dilation	5x10^{-3}mM

Supplementary Table 1: Model parameters for equations described in the main text. *: This value of g_r incorporates the cleft/perisynaptic volume ratio and the percentage of glutamate spilled to the perysinaptic space.

[18] C. Magri, U. Schridde, Y. Murayama, S. Panzeri, N. K. Logothetis, The amplitude and timing of the bold signal reflects the relationship between local field potential power at different frequencies, Journal of Neuroscience 32 (2012) 1395–1407.

[19] A. Belitski, A. Gretton, C. Magri, Y. Murayama, M. A. Montemurro, N. K. Logothetis, S. Panzeri, Low-frequency local field potentials and spikes in primary visual cortex convey independent visual information, Journal of Neuroscience 28 (2008) 5696–5709.

[20] G. R. Gordon, H. B. Choi, R. L. Rungta, G. C. Ellis-Davies, B. A. MacVicar, Brain metabolism dictates the polarity of astrocyte control over arterioles, Nature 456 (2008) 745–749.

[21] K. J. Friston, K. H. Preller, C. Mathys, H. Cagnan, J. Heinzle, A. Razi, P. Zeidman, Dynamic causal modelling revisited, Neuroimage 199 (2019) 730–744.

**Astrocyte calcium dynamics**	
τ_A	0.55s
O_M	0.05x10^2(mMs)$^{-1}$
χ	0.75
O_β	1x10^{-3}mM/s
O_δ	0.05e-3x10^{-3}mM/s
κ_δ	1x10^{-3}mM
O_{3K}	4.5x10^{-3}mM/s
K_δ	0.5x10^{-3}mM
K_D	0.5x10^{-3}mM
K_3	1x10^{-3}mM
Γ_{5P}	0.1s^{-1}
Γ_C	6s^{-1}
C_T	2x10^{-3}mM
ρ_A	0.18
Γ_L	0.1s^{-1}
O_P	0.9x10^{-3}mM/s
K_P	0.05x10^{-3}mM
d_1	0.13x10^{-3}mM
d_2	1.05x10^{-3}mM
d_3	0.9434x10^{-3}mM
O_2	0.2x10^3(mMs)$^{-1}$
Γ_2	1.05s^{-1}
**Balloon model**	
α_V	0.4
τ_{vf}	3s
a_{fout}	20
$Extr_o$	0.4

Supplementary Table 2: Model parameters for equations described in the Supplementary Information.
